# Targeting Non-Coding RNAs for the Development of Novel Hepatocellular Carcinoma Therapeutic Approaches

**DOI:** 10.3390/pharmaceutics15041249

**Published:** 2023-04-15

**Authors:** Tanja Jesenko, Simona Kranjc Brezar, Maja Cemazar, Alice Biasin, Domenico Tierno, Bruna Scaggiante, Mario Grassi, Chiara Grassi, Barbara Dapas, Nhung Hai Truong, Michela Abrami, Fabrizio Zanconati, Deborah Bonazza, Flavio Rizzolio, Salvatore Parisi, Giorgia Pastorin, Gabriele Grassi

**Affiliations:** 1 Department of Experimental Oncology, Institute of Oncology Ljubljana, Zaloska 2, SI-1000 Ljubljana, Slovenia; tjesenko@onko-i.si (T.J.); skranjc@onko-i.si (S.K.B.); mcemazar@onko-i.si (M.C.); 2 Faculty of Health Sciences, University of Primorska, Polje 42, SI-6310 Izola, Slovenia; 3 Department of Engineering and Architecture, Trieste University, via Valerio 6, I-34127 Trieste, Italy; alice.biasin@phd.units.it (A.B.); mario.grassi@dia.units.it (M.G.); michela.abrami@dia.units.it (M.A.); 4 Department of Life Sciences, Cattinara University Hospital, Trieste University, Strada di Fiume 447, I-34149 Trieste, Italy; tiernodomenico@gmail.com (D.T.); bscaggiante@units.it (B.S.); b.dapas@alice.it (B.D.); 5 Degree Course in Medicine, University of Trieste, I-34149 Trieste, Italy; chiara.grassi2@studenti.units.it; 6 Faculty of Biology and Biotechnology, VNUHCM-University of Science, Ho Chi Minh City 70000, Vietnam; thnhung@hcmus.edu.vn; 7 Department of Medical, Surgical and Health Sciences, University of Trieste, Cattinara Hospital, Strada di Fiume, 447, I-34149 Trieste, Italy; fabrizio.zanconati@asugi.sanita.fvg.it (F.Z.);; 8 Pathology Unit, Centro di Riferimento Oncologico di Aviano (CRO) IRCCS, I-33081 Aviano, Italy; flavio.rizzolio@unive.it; 9 Department of Molecular Sciences and Nanosystems, Ca’ Foscari University of Venice, I-30172 Venezia, Italy; salvatore.parisi@phd.units.it; 10 Doctoral School in Molecular Biomedicine, University of Trieste, I-34149 Trieste, Italy; 11 Pharmacy Department, National University of Singapore, Block S9, Level 15, 4 Science Drive 2, Singapore 117544, Singapore; phapg@nus.edu.sg

**Keywords:** hepatocellular carcinoma, lifer fibrosis, micro interfering RNAs, long non-coding RNAs, circular RNAs

## Abstract

Hepatocellular carcinoma (HCC) remains a global health challenge, representing the third leading cause of cancer deaths worldwide. Although therapeutic advances have been made in the few last years, the prognosis remains poor. Thus, there is a dire need to develop novel therapeutic strategies. In this regard, two approaches can be considered: (1) the identification of tumor-targeted delivery systems and (2) the targeting of molecule(s) whose aberrant expression is confined to tumor cells. In this work, we focused on the second approach. Among the different kinds of possible target molecules, we discuss the potential therapeutic value of targeting non-coding RNAs (ncRNAs), which include micro interfering RNAs (miRNAs), long non-coding RNAs (lncRNAs) and circular RNAs (circRNAs). These molecules represent the most significant RNA transcripts in cells and can regulate many HCC features, including proliferation, apoptosis, invasion and metastasis. In the first part of the review, the main characteristics of HCC and ncRNAs are described. The involvement of ncRNAs in HCC is then presented over five sections: (a) miRNAs, (b) lncRNAs, (c) circRNAs, (d) ncRNAs and drug resistance and (e) ncRNAs and liver fibrosis. Overall, this work provides the reader with the most recent state-of-the-art approaches in this field, highlighting key trends and opportunities for more advanced and efficacious HCC treatments.

## 1. Hepatocellular Carcinoma

Liver cancer remains a global health challenge and its incidence is still increasing [1]. The disease, representing the third leading cause of cancer deaths worldwide, is characterized by a poor prognosis [2]. Most diagnoses occur in subjects aged between 60 and 70 years, with men being predominantly affected [3]. Primary liver cancer is a malignant tumor that begins in the liver and encompasses different histological types. Hepatocellular carcinoma (HCC) is the most common type of primary liver cancer, which starts in the hepatocytes [4]. Other less common types are cholangiocarcinoma, which arises in the cells lining the bile ducts, and the very rare primary hepatic angiosarcoma, arising in hepatic blood vessels [4]. The major risk factors of HCC development include chronic alcohol consumption, hepatitis B or C infection, non-alcoholic fatty liver disease, non-alcoholic steatohepatitis, diabetes, exposure to aflatoxins and others [4]. Notably, over 90% of HCC cases occur in the scope of chronic liver disease, especially liver fibrosis (LF) from any etiology [5]. A number of molecular/cellular elements indicate a strict connection between LF and HCC [6]. In this regard, LF contributes to the formation of a “premalignant” environment [5] able to induce HCC development [7]. A very recent breakthrough paper [8] reported that hepatic stellate cells (HSCs) substantially contribute to HCC development. HSCs [9], which have the features of fibroblasts, are localized in the liver in the space of Disse [10]. Due to external pathological stimuli, quiescent HSCs trans-differentiate into proliferative and migratory myofibroblasts (cell activation), secreting extracellular matrix (ECM) protein, a hallmark of LF [9], resulting in progressive organ failure. We now know [8] that upon activation, HSCs also secrete HCC-promoting mediators, causing HCC occurrence in LF. 

The molecular landscape of HCC is heterogeneous as it varies according to distinct etiologies. Its pathogenesis is complex and involves alterations of several molecular pathways such as regulation of cell cycles, alteration of DNA methylation, chromosomal instability, immunomodulation, non-coding RNA dysregulation and others [11]. While the pathophysiology and drivers of the disease are being extensively investigated, treating HCC still remains challenging due to the molecular heterogeneity of tumors. Notably, approximately 25% of HCC tumors present actionable mutations; however, the prevalence of most mutations is less than 10%, thus complicating the search for targeted therapies [11]. 

### Available Therapeutic Approaches for HCC

Standard treatment options for HCC can be divided into three categories based on the disease stage. The curative options for early stage tumors encompass surgery, liver transplantation and ablation [4]. This approach is indicated for patients with single tumors and maintained liver function [12]. For ablation, microwave ablation (MWA) or radiofrequency ablation (RFA), both based on thermal ablation, are most commonly used, with MWA being more convenient for the treatment of larger lesions, especially those in close proximity to blood vessels [13]. In addition to thermal ablation, non-thermal ablation therapies, such as irreversible electroporation and electrochemotherapy, are also suitable for treatment of HCC, and can be performed either during open surgery, percutaneously or laparascopically [14,15]. For intermediate stage tumors, loco-regional therapies such as transarterial chemoembolization (TACE) and transarterial radioembolization (TARE) are utilized [4]. TACE is the standard of care for patients without curative treatment options whose disease is limited to the liver without macro-vascular invasion, or as a transitional treatment option for patients listed for liver transplantation. Surgical and loco-regional therapies have been reviewed extensively elsewhere [4,12]. For advanced stage tumors and for patients that do not qualify for local therapies, therapeutic options are limited. Systemic treatments, especially with conventional cytotoxic drugs, are usually ineffective [12]. Over the past three years, the rapid progress in molecular targeted therapies has dramatically changed the treatment landscape for advanced HCC. Immune checkpoint therapies are now being incorporated into HCC therapies, and their combination with molecular targeted therapy is emerging as a tool to enhance the immune response [16]. Obviously, first line and second line immune treatments are chosen based on the different stages of tumor progression. To date, first lines molecular therapies include the use of sorafenib and lenvatinib (multi-kinase inhibitors), atezolizumab (PD-L1 antibody) and bevacizumab (VEGF-A antibody). Second line therapies include regorafenib, cabozantinib (multi-kinase inhibitors) and ramucirumab, a recombinant monoclonal antibody that binds to and inhibits VEGFR-2 [12,17].

## 2. Development of Novel Targeted Therapies for HCC

Although great therapeutic advances have been made in recent decades, the prognosis for HCC patients remains poor due to late diagnosis, chemotherapy failure, frequent recurrence and lack of effective molecular targets. Thus, novel therapeutic approaches need to be developed. In this regard, we believe that for the development of optimized drug-based approaches, two strategies should be considered to improve effectiveness and safety. First, it is necessary to develop appropriate drug delivery systems able to drive the therapeutic molecule(s) to the diseased area without affecting the healthy surrounding tissues. A targeted delivery system has in principle the possibility to deliver a higher drug concentration at the tumor site, thus increasing effectiveness. Moreover, it has the advantage of preserving the normal cells and thus protecting the function of healthy tissue. Additionally, pato-physiological features specific to HCC could be exploited to develop selective HCC delivery systems [18]. The second aspect to be considered for the development of optimized therapeutic approaches for HCC consists of the choice of molecular target(s) whose expression is confined to tumor cells. This should guarantee high specificity of action, again leaving the normal cells unaffected. Such targets, however, may not be easily identifiable, as often they are also expressed in normal cells, although to a lower extent. We thus believe that in order to reach the highest level of specificity and effectiveness, novel therapeutic approaches for HCC should target both cancer cells and cancer-causing gene products. 

A discussion of cancer-cell-targeted delivery approaches is not the focus of this review as it has been described elsewhere [18,19,20,21,22,23]. Here, instead, we emphasize on research studies dealing with the identification of biological molecules causing HCC. Among the different kinds of molecules, we concentrate on the potential therapeutic roles of targeting non-coding RNAs (ncRNAs). The scientific interest in ncRNAs is demonstrated by the fact that in the last 20 years, the number of published papers passed from about 2200 in the year 2000 to about 25,000 in the year 2020 (source: PubMed, search performed on the 25 February 2023, key words: non-coding RNAs). To provide the reader with an updated description of the state-of-the-art approaches, we decided to focus on papers published in the last four years (2020–2023). The papers discussed have been subdivided into five sections, i.e., papers mainly describing (a) micro interfering RNAs (miRNAs), (b) long-non-coding RNAs (lncRNAs), (c) circular RNAs (circRNAs), (d) ncRNAs and drug resistance and (e) ncRNAs and liver fibrosis (LF). 

Milestones studies (reviewed in [24]) about ncRNAs in HCC are represented by (1) the discovery of the first liver specific miRNA (miR 122, 2005), (2) the first miRNA profiling in HCC (2006), (3) the discovery that ULCH lnRNA is upregulated in HCC (2007) and (4) the first lncRNA profiling in HCC (2011). For an overview of the previous works published in the field of ncRNAs and HCC before 2020, the readers can refer to two excellent reviews [24,25]. 

## 3. Non-Coding RNAs

So far, the most investigated types of ncRNAs are miRNAs, small interfering RNAs (siRNAs), lncRNAs and circRNAs [26].

### 3.1. miRNA and siRNA

MiRNA and siRNA are non-coding double-stranded RNAs approximately 22 nucleotides in length. While miRNAs are exclusively of endogenous origin, siRNAs can originate from exogenous sources such as invasive nucleic acids (viruses and transposons). However, siRNAs can be also of intracellular origin, being the products of different endogenous sources including pseudogene-derived transcripts [27] and lncRNAs. 

**Figure 1:**
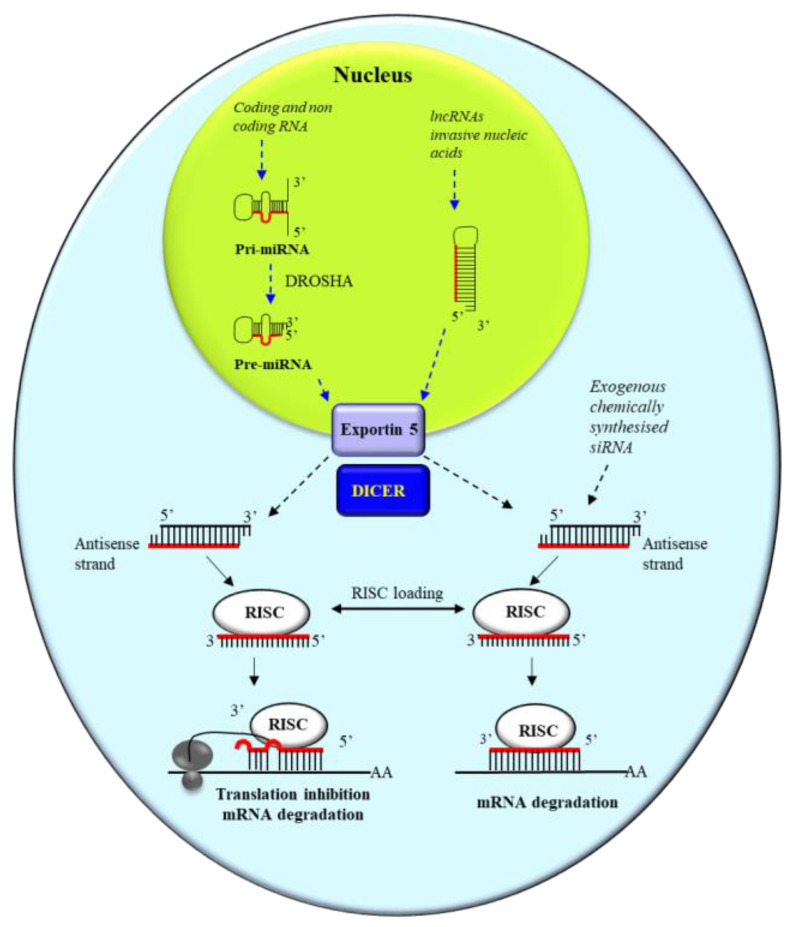
miRNAs originate from the transcription of a long precursor named pri-miRNA, processed to pre-miRNA by the enzyme Drosha. The pre-miRNA is then exported to the cytoplasm by means of Exportin 5, where the DICER enzyme generates a double-stranded mature RNA (miRNA). The antisense strand of the mature miRNA is loaded onto the enzymatic complex RISC and drives it to the target mRNA, inducing translation inhibition (imperfect base pairing with the target) or in some cases also mRNA degradation (perfect base pairing with the target). siRNAs follow the same pathway as miRNAs, except that they originate from lncRNAs/invasive nucleic acids and invariably induce target mRNA degradation.

MiRNA generation (Figure 1) begins in the cell nucleus with the transcription of a long precursor defined primary miRNA (pri-miRNA) [28], which in turn is processed by the enzyme Drosha [29]. The pre-miRNA is then exported to the cytoplasm by means of Exportin 5 (Exp5), where the DICER enzyme [27] generates a double strand RNA (mature miRNA) of approximately 22 nucleotides and bearing 2-nt 3’ overhangs [30]. The mature miRNA is loaded onto the enzymatic complex RISC (RNA-induced silencing complex) [27]. While the sense strand is discarded, the antisense strand allows the RISC recognition of the target mRNA that in turn undergoes translational repression [31]. For efficient translation repression, a contiguous and perfect base pairing of the first 2–8 nucleotides from the 5’ end of the antisense strand with the target is required [32]. A perfect complementarity is not required in the remaining nucleotides. However, when full complementarity occurs, the degradation of the target RNA takes place. SiRNA generation follows a similar pathway to that of miRNA. The siRNA antisense strand invariably requires a perfect complementarity with the target RNA to induce the degradation, as a single mismatch can profoundly impair target RNA degradation by RISC [33]. While naturally occurring siRNAs act against invading exogenous nucleic acid, they can be also of exogenous origin, being chemically synthesized and introduced into a given cell type to target deleterious cellular RNAs. miRNAs regulate the expression of genes involved in many different cellular pathways. Notably, a single miRNA can target different cellular RNAs, and a given cellular RNA can be targeted by multiple miRNAs. For these reasons, miRNA activity is defined as “promiscuous”. The promiscuous action of miRNAs and the interaction with lncRNAs/circRNAs (see below) outline the complexity of gene expression regulation by ncRNAs.

### 3.2. lncRNAs

**Figure 2:**
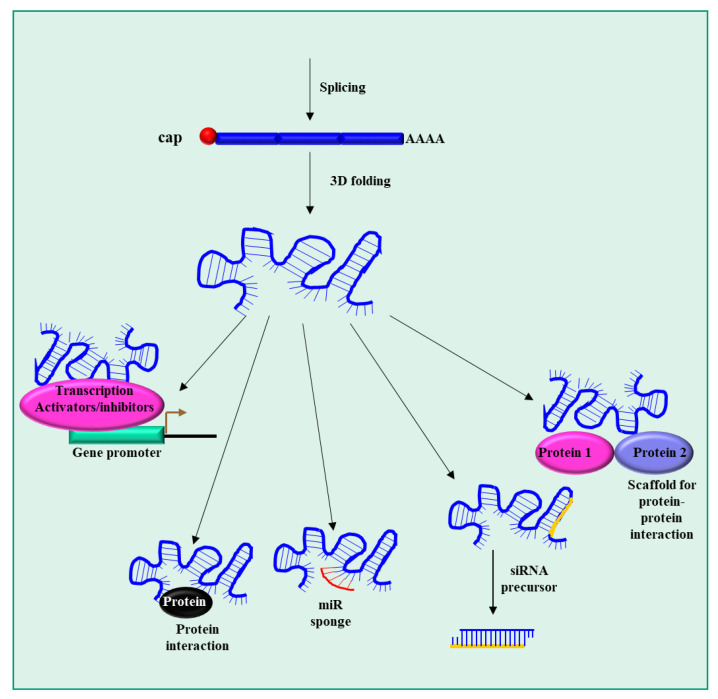
Most long, non-coding RNAs (lncRNAs) undergo splicing, capping and polyadenylation. Once transcribed, lncRNAs adopt a complex 3D structure which is eventually responsible for their biological effects that include the regulation of transcription via the recruitment of transcription activators/repressors to the promoters of their target genes; the binding to proteins, thus preventing their functions; the sponging of miRNA; the possibility to act as precursors for small interfering RNAs; and the ability to act as scaffolds to promote the formation of protein complexes.

LncRNAs are constituted by a single-stranded RNA molecule with a length arbitrarily set longer than 200 nucleotides [34]. The biogenesis (Figure 2) of lncRNAs substantially overlaps that of mRNAs, as most of them undergo splicing, capping and polyadenylation. They are transcribed by RNA polymerase II (Pol II). The only clear difference from mRNA is that they do not have a translated open reading frame. Additionally, compared to mRNAs, they tend to be shorter, contain fewer but longer exons and are expressed at low levels [35]. Moreover, lncRNAs in general exhibit more tissue-specific expression profiles than mRNAs [35].

lncRNAs can be found in genic and intergenic regions; moreover, while they can be transcribed from the plus DNA strand, lncRNAs transcribed from the minus strand in the opposite direction from protein-coding genes also exist [36]. The complex biological role of lncRNAs stems from their articulated 3D structure, which depends on the linear base sequence. Thanks to the 3D structure, lncRNAs have the ability to perform multiple functions [37], among which are (1) regulation of transcription via the recruitment of transcription activators/repressors to the promoters of their target genes; (2) binding to transcriptional-related proteins, thus preventing their functions; (3) binding (sponge effect) to miRNA, thus impairing their activities; (4) the possibility to act as precursors for some small interfering RNAs; and (5) the ability to act as scaffolds to promote the formation of protein complexes. Due to the complex and multiple biological roles of lncRNAs, it is not surprising that their altered expression and/or mutation are implicated in numerous human diseases [38].

### 3.3. circRNAs

**Figure 3:**
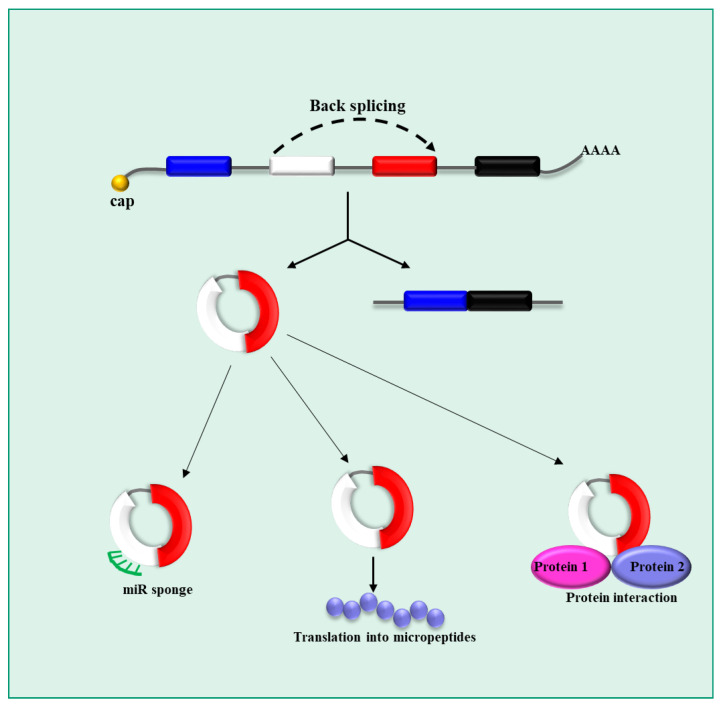
Although circRNAs have multiple biogenesis mechanisms, a common one is represented by back-splicing. This process seems to require canonical spliceosomal machinery with cis- and trans-regulatory elements. Back-splicing can be induced by protein dimerization, sequence complementarity of flanking introns and exon skipping mechanisms. After formation, circRNAs are exported into the cytoplasm, where they can sponge miRs, can be translated into micropeptides or can function as substrates for protein–protein interactions.

circRNAs are constituted by single-stranded transcripts covalently closed to form a circular structure [39]. Due to this structure, they are more stable than other RNA types as they are more resistant to degradation. circRNAs can be generated by RNA polymerase II (Pol II) [40] from precursor mRNA (pre-mRNA) (Figure 3). Notably, multiple circRNAs can be generated from the same gene by alternative splicing [41]. There does not seem to be a clear correlation between circRNA expression levels and those of their corresponding mRNAs [42]. Once generated, circRNAs are exported into the cytoplasm. The generation process of circRNAs, called back-splicing, is not fully understood yet. It seems to require canonical spliceosomal machinery with cis- and trans-regulatory elements; the former are substantially represented by the splice site sequences (no other specific exonic sequence seems to be necessary) and the latter are represented by RNA binding protein [42]. The length of circRNAs ranges from a minimum of 30–40 nucleotides to longer sequences that seem to enhance circRNA production [43]. Although ciRNAs may contain multiple exon sequences, two–three exons are typically present in their sequence [43]. Interestingly, circRNAs containing intronic sequences have been described as well. Even though, in general, the expression levels of circRNAs are rather low [44], circRNAs expressed abundantly have been reported [45]. Finally, for some cirRNAs, a tissue-specific expression has been described. With regard to the biological role of circRNAs, it has been proposed that they function as miRNA sponges in the cytoplasm. Thus, they inhibit miRNA-dependent control of gene expression. Moreover, some circRNAs can be translated into micropeptides or can function as substrates for protein–protein interactions.

## 4. Therapeutic Potential of ncRNA Targeting

As will be detailed in the following sections on the role of ncRNAs in HCC and LF, the effects of ncRNAs are very complex and only partially understood. Thus, it is not possible to draw a general simplified picture of their mode of action and interconnection. However, with the aim to simplify the complex scenario, we have subdivided ncRNAs as “oncogenic“, when they promote cancer, or as “tumor suppressors“, when they prevent cancer development. In the first case, they typically inhibit tumor suppressor molecules able to downregulate cell proliferation/migration or to promote cell apoptosis. In the second case, ncRNAs downregulate pro-proliferative/migratory molecules and/or inhibit anti-apoptotic molecules. To easily understand the oncogenic/tumor suppressor nature of the ncRNAs presented here, in each table reassuming the ncRNAs described, we have reported an oncogenic (O) or a tumor suppressor (Ts) role. With regard to the potential therapeutic strategies for “O” ncRNAs, the prevention of their effects is mainly based on the silencing by siRNAs. This aspect is clearly shown by the papers described in Section 4.1,Section 4.2,Section 4.3 and Section 4.4. For “Ts” ncRNAs, their overexpression might be induced in cancer cells, even though this approach may be rather complex due to the limited technical knowledge in this field. 

### 4.1. miRNAs

All the works presented in this section are summarized in Table 1.

The expression level of miRNAs, similar to many other genes, is regulated, among other mechanisms, by histone deacetylases (HDACs). HDACs can remove acetyl groups on N-terminal lysine residues of histones, resulting in a condensed chromatin structure with the consequent transcriptional repression. This kind of gene expression control falls within epigenetic mechanisms. Huge et al. [46] observed that in cultured HCC cell lines (HuH7, HepG2 and Hep3B), HDAC inhibition induced the expression of miR-129-5p. Notably, miR-129-5p downregulation is often observed in solid tumors such as HCC [57]. The transfection of miR-129-5p in cultured HCC cell lines (HLE, HLF, HuH7 and HepG2) resulted in decreased cell viability and also in a reduction in cell migration in HLE cells. These observations were confirmed in a xenograft mouse model of HCC (generated with the HuH7 cell line), where miR-129-5p reduced tumor growth. By bioinformatics and experimental tests, the transcription factor SOX4 (SRY-related HMG-box), involved in the regulation of TGF β1 (transforming growth factor-β1)-mediated epithelial-mesenchymal transition (EMT), was found to be a miR-129-5p target. TGF-β1 is a pleiotropic cytokine that regulates cell growth, differentiation, migration and apoptosis. The hepatoma-derived growth factor (HDGF), which has mitogen activity and increased expression in a variety of cancers, has been identified as an additional target of miR-129-5p. Notably, HDGF overexpression negatively correlated with HCC patient survival and its expression increased with the de-differentiation of HCC. Moreover, in vitro, its silencing reduced cell viability and migration and increased apoptosis. Together, the above data suggest that reactivation of miR-125-5p expression may be beneficial for HCC patients.

**Figure 4:**
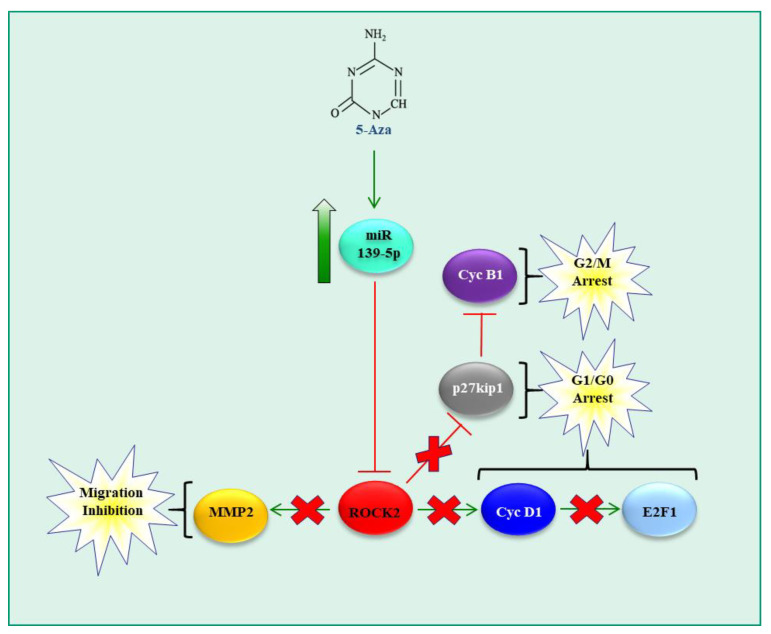
miR-139-5p, Tonon et al. [47]. The demethylating drug 5-azacytidine reactivates the expression of miR-139-5p, which in turn downregulates the translation of the Rho-associated coiled-coil kinase-2 (ROCK2). ROCK2 targeting prevents the activation of the pro-proliferative proteins cyclin D1 (CycD1) and E2F1. Additionally, ROCK2 targeting relieves its inhibition on the cell cycle inhibitor p27^kip1^ which can downregulate the effects of the pro-proliferative protein cyclin B1 (Cyc B1). Finally, ROCK2 targeting abolishes the activation of the pro-migratory protein matrix metalloproteinase 2 (MMP2).

Another epigenetic mechanism that regulates gene and miRNA expression relies on the methylation of 5′-CpG-3′ palindromes mostly present in human gene promoters. Following methylation operated by DNA methyltransferases, other proteins containing a methylcytosine-binding domain [58,59] are recruited to the methylation sites. This results in gene transcription repression, as transcription factors do not have access to the gene promoter. Recently, we studied [47] the effects of 5-azacytidine (5-Aza), a drug with the ability to revert pathological promoter methylation, on HCC cell lines. Among many other effects, we observed the reactivation of the expression of miR-139-5p. miR-139-5p is located within the sequence of the phosphodiesterase 2A (PDE2A) gene [59,60]. miR-139-5p expression is reduced in HCC tissue compared to adjacent non-tumor tissue and its level inversely correlates with patient outcomes [61]. In the human HCC cell lines JHH6/HuH7 and in rat N1-S1, we showed that, upon reactivation of miR-139-5p expression by 5-Aza, HCC cells reduced viability, growth, migration and adhesion. At the molecular level, this was due to Rho-associated coiled-coil kinase-2 (ROCK2) targeting by miR-139-5p. ROCK2 is a kinase able to phosphorylate different proteins by modifying the assemblies of filamentous actin and eventually regulating cell contractility, motility and morphology. ROCK2 downregulation determined the inhibition of different downstream targets such as the pro-proliferative proteins cyclin D1 and E2F1 and the pro-migratory protein matrix metalloproteinase 2. Additionally, we observed the upregulation of the cell cycle inhibitor p27^kip1^ which, in turn, inhibited the pro-proliferative protein cyclin B1 (Figure 4). The in vitro data were confirmed in multiple in vivo HCC models (a mouse xenograft mouse model, an orthotopic rat model and a xenograft zebrafish model of HCC). Our data suggest that demethylating agents may be of therapeutic interest, as they can reactivate the expression of HCC suppressor molecules such as miR-139-5p.

Many HCCs are caused by hepatitis B virus (HBV) infection. Thus, Gao et al. [48] explored the role of hepatitis B virus X protein (HBx) in HCC development. As the HCC cell line MHCC97H expresses high levels of HBx, it was chosen for in vitro study. HBx silencing by siRNA induced a significant reduction in cell growth and colony formation. This observation was confirmed in a xenograft mouse model where tumor formation was significantly delayed. By an miR microarray, the upregulation, among others, of miR-137 in MHCC97H treated by siRNA anti HBx compared to control siRNA-treated cells was observed. miR-137 was chosen due to its known involvement in HCC [62]. The authors proved that HBx downregulation resulted in miR-137 expression via the induction of promoter methylation, as shown by the fact that MHCC97H treatment by 5-Aza restored miR-137 expression. Notably, miR-137 overexpression reduced cell growth both in vitro and in vivo. Evidence was also provided that Notch-1 was the target of miR-137 and that Notch downregulation resulted in impaired cell proliferation. Notch-1 is a transmembrane protein with different biological roles, including the regulation of the interactions between physically adjacent cells. These data, together with our above reported mechanisms [47], underline the relevance of miR promoter methylation in HCC, suggesting the significance of demethylating agents in HCC.

In recent years, miR-142-3p has gained attention in the HCC field as its reduced levels are associated with disease progression. In this regard, it has been shown that circRNA WHSC1 (CircWHSC1) sponges miR-142-3p, thereby increasing the levels of its target homeobox A1 (HOXA1), which promotes HCC (see also Section 4.3: circRNAs) [63]. Moreover, lncRNA712 can target the miR-142-3p/Bach-1 pathway, promoting HCC development (see also Section 4.2: lncRNAs) [64]. Zeng et al. [49] further studied the role of miR-142-3p in HCC, confirming that its levels are reduced in HCC and that patients with higher expression levels have longer survival. In cultured HCC cell lines (Huh7 and HCCLM3), miR-142-3p transfection reduced the proliferation rate and promoted cell apoptosis. Surprisingly, however, cell migration and invasion were augmented. In a xenograft mouse model of HCC obtained by inoculating HuH7, it was confirmed that miR-142-3p overexpression reduced the tumor size. However, no data about animal survival were reported. By bioinformatics analyses and experimental confirmation, it was shown that a possible target for miR-142-3p was phosphatidylinositol-4,5-bisphosphate 3-kinase catalytic subunit gamma isoform (PIK3CG). This is a modulator of extracellular signals, such as those stimulated by E-cadherin-mediated cell–cell adhesion. Its level is increased in prostate cancer and its targeting reduces cancer cell growth. Thus, the data of Zeng et al. [49] further point toward the importance of increasing the level of miR-142-3p in HCC. 

Through a database search, Kannan et al. [50] reported that astrocyte elevated gene-1 (AEG-1) is upregulated in HCC, and that patients with low expression rates have a longer survival compared to those with a high expression. AEG-1 is overexpressed in different tumor types, where it acts as an oncogene favoring metastasis, invasion, migration and chemo-resistance. Similarly, miR-221 has been found to be upregulated in HCC and to inversely correlate with patient prognosis. The above observations were confirmed in a panel of HCC cell lines (HepG2, HuH7 and Hep3B), where the pro-proliferative, pro-migratory and anti-apoptotic effects of miR-221 were reported. Whereas miR-221 silencing/overexpression did not alter AEG-1 expression, the silencing of AEG-1 resulted in a marked decrease in miR-221 levels. These data suggest that AEG-1 is not a target of miR-221, while AEG-1 regulates miR-221 expression. Unfortunately, no mechanistic explanation was provided for this last observation. The authors also showed that AEG-1 or miR-221 silencing resulted in the downregulation of a number of cell-cycle-promoting genes and in the upregulation of anti-proliferative genes such as p53. Moreover, the downregulation of matrix metalloproteinase 9 (MMP9) was observed, which belongs to zinc-metalloproteinase family involved in the degradation of the extracellular matrix that favors cell migration and invasion. The fact that the panel of up- and down-regulated genes was identical following both AEG-1 and miR-221 silencing seems to confirm the concept that AEG-1 exerts its effect via miR-221 upregulation. It remains to be determined whether this is a direct or indirect effect. 

Protein tyrosine phosphatase non-receptor type 12 (PTPN12) is a member of the protein tyrosine phosphatase (PTP) family, regulating cell growth, differentiation and migration. Liang et al. [51] observed that PTPN12 was downregulated in the HCC tissue of 45 HCC patients compared to the corresponding adjacent tissues. Moreover, the expression of PTPN12 was associated with a larger tumor size and the extent of lymph node metastasis. In vitro, PTPN12 silencing resulted in increased cell growth in SMMC7721 and MHCC97H cell lines, as well as migration and invasion. In parallel, the downregulation of E-cadherin and the upregulation of Vimentin were observed, markers of EMT triggering. The authors demonstrated that PTPN12 is the target of miR-106a-5p. The inhibition of miR-106a-5p resulted in the repression of SMMC7721and MHCC97H growth, migration, invasion and EMT progression. Despite being elegantly conducted, this work needs to be confirmed through an in vivo model to fully define the relevance of miR-106a-5p targeting.

Liu et al. [52] observed that the expression level of miR-29b-3p was reduced in hepatoma 22 (H22) cells obtained from ascites tumor-bearing mice compared to the control. Additionally, overexpression of miR-29b-3p resulted in the downregulation of cell proliferation and in an increase in cell apoptosis. At the molecular level, the authors showed that miR-29b-3p overexpression was accompanied by increased expressions of TGF-β1 and p53. TGF-β1 regulates cell growth, differentiation, migration and apoptosis. Notably, TGF-β1 can improve p53 expression, triggering apoptosis of hepatic stellate cells. P53 is a transcription factor that negatively regulates the cell cycle and functions as a tumor suppressor gene. Despite being of potential interest, these data lack the evidence of a mechanistic connection between miR-29b-3p and TGF-β1/p53 and need to be confirmed in a more sophisticated in vivo model of HCC.

Zhang et al. [53] showed that miR-495 levels were lower in HCC tissue samples (from 118 patients) compared to adjacent non-neoplastic tissues and that its levels were directly correlated with patient survival. This observation was confirmed in the HCC cell lines HepG2 and HCCLM7 compared to HL-7702 and THLE3, i.e., two normal human liver cell lines. The transfection of miR-495 into the HCC cell lines HepG2/HuH7 reduced cell viability, increasing the number of cells in the G1/G0 phase and reducing the number of cells in the S and G2/M cell cycle phases. Moreover, senescence was induced, as evaluated by the increase in the senescence marker SA-β-gal. Afterwards, it was proven that C1q tumor necrosis factor (TNF)-related protein (CTRP3) is a target of miR-495. CTPR3 has regulatory effects on cell growth and/or differentiation in various cells. Notably, CTRP3 levels were found to be increased in HCC tissues, indicating the inverse relation with miR-495. In a xenograft mouse model of HCC obtained by injecting the HCC cell line HepG2 subcutaneously, it was observed that miR-495 overexpression significantly delayed tumor growth. This was accompanied by a decrease in the expression of CTRP3. These data indicate the significance of increasing miR-495 levels to fight HCC.

Xiang et al. [54] observed that the expression levels of miR-23a-3p were upregulated in HCC tissues (30 samples) compared to the adjacent normal liver tissue. The opposite behavior was detected for Protocadherin17 (PCDH17), a tumor suppressor gene involved in the control of cell cycles. Among HCC cell lines, Hep3B exhibited the lowest miR-23a-3p expression, while HepG2 had the highest expression. Transfection of miR-23a-3p in Hep3B promoted cell proliferation, with an increase in S phase cells and a decrease in G1 phase cells; this was in line with the increased expression of the G1/S promoting proteins cyclin D1 and cyclin E and the decrease in the anti-proliferative protein p27^kip^. The opposite effect was observed in HepG2 transfected with an anti-miR-23a-3p. Together, these observations point towards the oncogenic role of miR-23a-3p. At the molecular level, the authors showed that PCDH17 was the target of miR-23a-3p. Moreover, in vivo, in an HCC xenograft model generated with HepG2, the inhibition of miR-23a-3p reduced tumor cell growth and upregulated PCDH17. While these data are of interest, the connection between miR-23a-3p/PCDH17 and cylinD1/cyclin E/ p27^kip1^ remains to be clarified.

Zhao et al. [55] showed that miR-126-5p was downregulated in the HCC cell lines Hep3B, Huh7, MHCC97H and HCCLM3 in comparison with LO2, which are considered normal hepatic cells. However, caution should be used with this cell type as it has been shown to derive from the cervical cancer line HeLa [65]. miR-126-5p downregulation observed in HCC cell lines was then confirmed in 18 HCC tissue samples compared to the adjacent non-tumor tissue. miR-126-5p transfection into Hep3B and MHCC97H significantly reduced cell viability, while its inhibition (via an anti-miR) greatly stimulated cell viability. miR-126-5p transfection also impaired cell migration, invasion and colony formation. Additionally, epidermal growth factor receptor (EGFR) was found to be the target of miR-126-5p. EGFR overexpression is related to uncontrolled cell growth and thus to the genesis of different human cancers. EGFR overexpression or silencing resulted in increased or decreased cell growth/migration, respectively. These interesting data should be consolidated in in vivo HCC models, together with a description of a mechanistic connection between EGFR and cell proliferation/migration.

While it is known that miR-642a is implicated in HCC, most of the molecular mechanisms responsible for its effect are not known [66]. Yu et al. [56] investigated the role of miR-642a in HCC, showing that it is upregulated in HCC tissues (60 patients) compared to adjacent non-tumor tissues. Then, it was shown that Semaphorin 4C (SEMA4C) is a target of miR-642a. SEMA4C is known to promote terminal myogenic differentiation in a p38-dependent manner. Notably, SEMA4C expression inversely correlates with that of miR-642a in HCC tissues compared to control tissues. In the HCC cell line HuH7, the authors showed that miR-642a downregulation reduced cell invasion and migration. Similar effects were observed following SEMA4C silencing by siRNAs. Finally, it was observed that miR-642a overexpression or SEMA4C silencing resulted in the impairment of the activity of p38 mitogen-activated protein kinases (p38-MAPK), a class of protein kinases involved in cell differentiation, apoptosis and autophagy. Unfortunately, a mechanistic link between miR-642a/SEMA4C and p38-MAPK was not provided; moreover, no in vivo data were presented to corroborate the in vitro observations.

### 4.2. lncRNAs

All the works presented in this section are summarized in Table 2.

As reported (see Section 4.1: miRNAs and siRNAs and 4.3: circRNAs), miR-142-3p has gained attention in the HCC field [49,63]. Cui et al. [64] showed that lncRNA lnc712 sponges miR-142-3p. Additionally, lnc712 expression levels are upregulated in HCC tissues (64 patients) compared to adjacent non-tumor tissue and its expression negatively correlates with patient prognosis. In vitro, overexpression of lnc712 promoted proliferation, migration and invasion of the HCC cell line SNU-182 compared to control. The opposite results were observed following miR-142-3p overexpression. The sponge effect of lnc712 on miR-142-3p was also shown, resulting in the upregulation of Bach-1, a target of miR-142-3p. Bach-1 is a transcription regulator protein that facilitates protein–protein interactions. Finally, in a xenograft mouse model of HCC, lnc712 silencing resulted in reduced tumor growth; however, no data about improved animal survival were provided.

lncRNA ASAP1-IT1 (the intronic transcript 1 (IT-1) of ArfGAP with SH3 domain, ankyrin repeat and PH domain 1 (ASAP1)) is known to correlate with the overall survival of ovarian cancer patients [78]. Liu et al. [67] reported lncRNA ASAP1-IT1 upregulation in HCC tissue samples (54 patients) compared to matched histologically normal liver tissues. Moreover, an inverse correlation between lncRNA ASAP1-IT1 levels and patient overall survival was observed. In vitro, in the HCC cell lines HuH7 and HepG2, the upregulation of lncRNA ASAP1-IT1 was observed in comparison with the non-tumorigenic LO2 hepatocyte cell line. In HepG2 and HuH7, the siRNA-mediated silencing of lncRNA ASAP1-IT1 resulted in a significant downregulation of cell viability, proliferation and migration. From the mechanistic point of view, it was shown that ASAP1-IT1 acts as a sponge for miR-221-3p. Unfortunately, neither the targets for miR-221-3p nor the effects of miR-221-3p overexpression have been investigated. Further studies are therefore necessary to fully understand the functional role of lncRNA ASAP1-IT1/miR-221-3p in HCC.

Long intergenic non-coding RNA 152 (LINC00152) is significantly upregulated in human HCC as a consequence of promoter hypomethylation [79]. Recently, Pellegrino et al. [68] reported that in HuH7 and HepG2 cells, LINC00152 silencing resulted in the reduction in cell viability and the colony formation capacity. Additionally, the authors showed that LINC00152 sponges miR143a-3p and that this effect was accompanied by the increased expression level of Kinesin Light Chain 2 (KLC2), Serine/Threonine Kinase 39 (STK39) and PHD Finger Protein 19 (PHF19). As STK39 and PHF19 have already been shown to have tumorigenic effects in HCC, attention was given to KLC2, a microtubule-associated protein that plays a role in organelle transport. siRNA-mediated silencing of KLC2 significantly reduced HepG2/HuH7 viability, the ability to form colonies and also migration in HuH7. In vivo, in 50 HCC sample tissues, LINC00152 and KLC2 expressions were upregulated compared to non-tumor surrounding liver tissue; in contrast, miR-143a-3p expression was downregulated. Together, these data support the concept of a relevant role of the LINC00152/miR143a-3p/ KLC2 axis in HCC and that its targeting may be of interest.

lncRNA Cancer Sensitivity 9 (CASC9) has been found to be related to different human cancers, including HCC [80]. Moreover, miR-424-5p has been shown to be implicated in the development of HCC [81]. Recently, Yao et al. [69] explored the connection between CASC9 and miR-424-5p in HCC. CASC9 was found to be overexpressed in HCC tissues (50 samples) compared to adjacent normal tissues. Moreover, its expression levels inversely correlated with patient prognosis. The opposite behavior was instead observed for miR-424-5p. In vitro, CASC9 was overexpressed in the HCC cell lines PLC/PRF/5, SNU-387, SNU-423 and SK-Hep1 compared to the control human liver epithelial cell lines (THLE-2). CASC9 silencing by siRNA reduced cell proliferation, colony formation and migration and promoted apoptosis in SK-Hep1 and PLC/PRF/5. Finally, the authors indirectly proved that CASC9 sponges miR-424-5p. Despite that no possible targets for miR-424-5p were provided in the present work, the presented data, together with those published before [80,81], support the rationale to target CASC9/miR-424-5p in HCC.

While the lncRNA LINC00839 is known to be upregulated in breast cancer [82], no information is available for HCC. By bioinformatic analyses and quantification in 60 HCC tissues, Zhou et al. [70] demonstrated that LINC00839 is upregulated compared to normal liver tissue and that its expression inversely correlates with patient overall survival. An increased expression was also observed in the HCC cell lines HuH6, HuH7, SK-hep1, HepG2 and PLC5 compared to LO2, a normal hepatocyte cell line. LINC00839 silencing by siRNA resulted in a marked downregulation of proliferation and migration/invasion and in an upregulation of apoptosis in HepG2 and PLC5 cell lines. By bioinformatic analyses, it was shown that LINC00839 interacts with miR-144-3p by sponging it. Notably, miR-144-3p is known to be a tumor suppressor in different types of human tumors. The relation of LINC00839 with miR-144-3p was also confirmed at the experimental level; LINC00839 silencing induced an increase in miR-144-3p levels and, in HCC tissues, the levels of LINC00839 and miR-144-3p were inversely correlated. WT1-associated protein (WTAP) was identified by both bioinformatic analyses and experimental tests as the target of miR-144-3p. WTAP is a protein known to facilitate the progression of HCC [83]. Together, these data, which need to be confirmed in an in vivo model of HCC, indicate that LINC00839/miR-144-3p/ WTAP targeting may be pursued in HCC.

lncPVT1 (plasmacytoma variant translocation 1) has been implicated in HCC development [84]. However, it is not known which factors control lncPVT1 expression and which are its targets. By experimental tests and bioinformatics, Xiong et al. [71] showed in the HCC cell lines SMMC-7721 and HepG2 cells that the oncoprotein c-Myc can bind to lncPVT1 promoter, inducing its transcription. By a similar approach, it was also shown that the tumor suppressor gene product p53 can bind to the lncPVT1 promoter, repressing its transcription. Furthermore, the authors observed that there was a direct correlation between lncPVT1 and c-Myc expression in 26 HCC samples, while the inverse correlation occurred with p53. Again, by experimental tests and bioinformatics, the authors proved that miR-214 targets lncPVT1 in HCC cell lines, reducing its levels. Notably, miR-214 was downregulated in HCC samples compared to non-tumor adjacent tissue. In SMMC-7721 and HepG2, miR-214 overexpression downregulated cell growth, migration and invasion. This observation was confirmed in a xenograft mouse model of HCC generated with HepG2 cells, where miR-214 overexpression or lncPVT1 inhibition significantly reduced tumor growth. Finally, gene product GDF15 (growth/differentiation factor 15), a known promoter of HCC [85], was found to be the downstream target of lncPVT1/miR-214 in HCC cells. miR-214 overexpression or lncPVT1 silencing by siRNAs resulted in the downregulation of the expression of GDF15 in SMMC-7721 and HepG2 cell lines. While these data are of interest, it remains unclear whether GDF15 downregulation depends on the direct effect of miR-214 or if it is a consequence of lncPVT1 downregulation by miR-214 or a combination of both.

**Figure 5:**
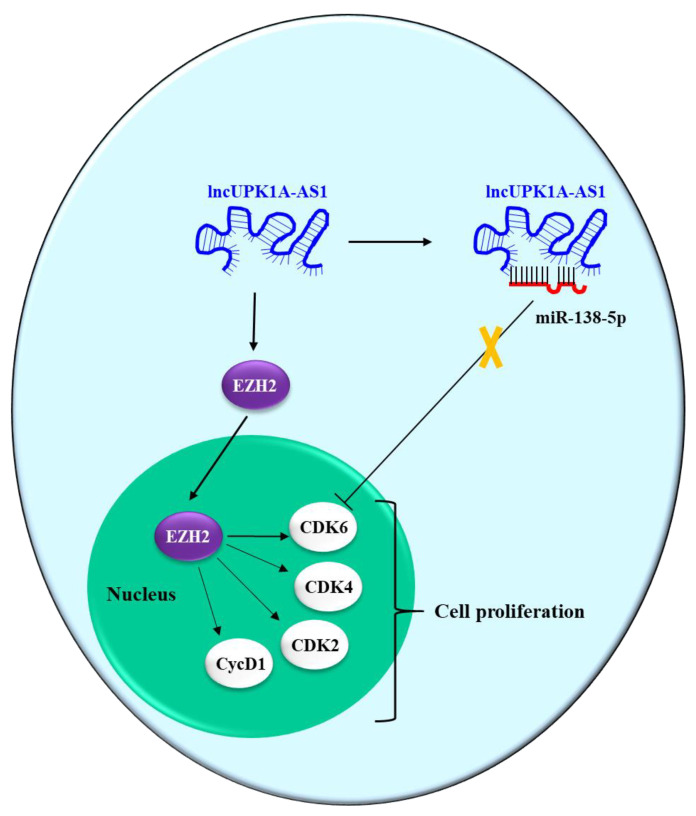
lncUPK1A-AS1, Zhang et al. [72]. This lncRNA promotes cell proliferation by favoring the migration of enhancer of zeste homolog 2 (EZH2) into the nucleus, where it promotes the expression of the pro-proliferative proteins CyclinD1 (CycD1) and cyclin-dependent kinases 6 (CDK6), 4 (CDK4) and 2 (CDK2). Moreover, lncUPK1A-AS1 promotes cell proliferation by sponging miR-138-5p, which can then no longer target CDK6.

UPK1A antisense RNA 1 (UPK1A-AS1) is a recently discovered lncRNA whose biological role has only been minimally identified. It is known that it is downregulated in esophageal squamous cell carcinoma (ESCC), and that it can downregulate proliferation, migration and invasion of ESCC cells by sponging microRNA-1248 [86]. Zhang et al. [72] studied the role of UPK1A-AS1 in HCC, showing that it is highly expressed in HCC cells compared to the human hepatocyte cell line LO2. Moreover, its downregulation reduced the proliferation of the HCC cell lines SK-Hep-1 and MHVV-97H, diminishing the number of cells in the S phase of the cell cycle and increasing the number in the G1 phase. In line with this observation, decreases in the levels of cyclin D1, CDK2, CDK4 and CDK6 were detected, which are all promoters of the G1/S phase transition. Moreover, UPK1A-AS1 silencing promoted apoptosis. These data were confirmed in a xenograft model of HCC. The authors also showed that UPK1A-AS1 physically interacts with the enhancer of zeste homolog 2 (EZH2). This is a histone-lysine N-methyltransferase enzyme that takes part in histone methylation and, eventually, in transcriptional repression. While UPK1A-AS1 did not affect EZH2 expression, it favored its translocation to the nucleus, thus promoting the expression of the G1/S phase transition regulators (cyclin D1, CDK2, CDK4 and CDK6). Hence, UPK1A-AS1 promoted cell proliferation via EZH2. As an alternative mechanism of action, it was shown that UPK1A-AS1 sponges miR-138-5p, which counts CDK6 among its targets. Thus, by sponging miR-138-5p, UPK1A-AS1 induces an increase in CDK6 and promotes cell growth (Figure 5). Finally, it was shown that UPK1A-AS1 and EZH2 are overexpressed in HCC tissues compared to the adjacent non-tumor tissues and that their expression levels inversely correlate with patient prognosis.

Zhao et al. [73] demonstrated that the lncRNA metastasis-associated lung adenocarcinoma transcript 1 (MALAT1) is overexpressed in HCC tissues (20 samples) compared to the adjacent non-tumor tissue. Moreover, it is overexpressed in the HCC cell line HepG2. Interestingly, it was shown that in spheroids generated from either HepG2 or HuH7, MALAT1 silencing significantly reduced sphere formation and size. This was paralleled by a reduction in the expression of different transcription factors responsible for cancer stemness such as the oncogene c-Myc. The authors also showed that MALAT1 sponges miR-375. One of the miR-375 targets was found to be yes-associated protein 1 (YAP1), a transcription regulator promoting the transcription of genes involved in cellular proliferation and suppressing pro-apoptotic genes. In conclusion, the authors proposed that MALAT1 increases the activity of YAP by sponging miR-375, resulting in increased cell proliferation.

The lncRNA linc00467 has been implicated in the regulation of the migration of lung adenocarcinoma cells [87]. Zheng et al. [74] investigated its role in HCC, showing its overexpression in HCC tissues (20 patients) compared to the adjacent non-tumor tissues. Moreover, linc00467 was found to be overexpressed in the HCC cell lines Bel-7402, SMMC-7721, HepG2, Hep3B and HCCLM3 compared to the non-tumorigenic liver cell line LO2. linc00467 silencing in SMMC-7721 and HepG2 significantly reduced cell viability, colony formation and migration, while increasing the apoptotic rate. Additionally, it was found that linc00467 sponges miR-18a-5p and that neural precursor-cell-expressed developmentally downregulated protein 9 (NEDD9) was the target of miR-18a-5p. Notably, NEDD is upregulated in HCC and is implicated in tumor metastasis [88]. Thus, following confirmation in an in vivo model of HCC, the targeting of linc00467–miR-18a-5p–NEDD9 may be of interest.

The oncogenic potential of long non-coding RNA 00958 (LINC00958) was first identified in bladder cancer [89] as well as in other cancer types. However, its involvement in HCC remains unknown. Zuo et al. [75] explored the significance of LINC00958 in HCC, showing, by both a database search and experimental tests (80 HCC patients), that this lncRNA is overexpressed in HCC tissue compared to the adjacent non-tumor tissue. Additionally, the expression level of LINC00958 inversely correlates with patient prognosis. LINC00958 upregulation was also observed in six HCC cell lines (Hep3B, HepG2, HuH7, MHCC-97H, Focus and HCCLM3) compared to the normal human liver cell line QSG-7701. Silencing of LINC00958 in HCCLM3 and Focus cell lines resulted in a reduction in proliferation, colony formation, migration and invasion. The authors identified miR-3619-5p as the target of LINC00958. Additionally, hepatoma-derived growth factor (HDGF) was shown to be the target of miR-3619-5p. HDGF is known to promote hepatoma cell growth. In a patient-derived xenograft (PDX) mouse model of HCC, the authors observed that LINC00958 overexpression promotes tumor growth, while its silencing downregulates tumor growth. This last observation was paralleled by the reduction in the expression of HDGF. Finally, the authors demonstrated that the systemic delivery of anti-LINC00958 siRNA prolonged animal survival, thus fully proving the potential therapeutic value of LINC00958 targeting.

Whereas it is known that epoxide hydrolase 1 (EPHX1) is implicated in HCC, the regulation of its expression by non-coding RNAs has not been defined. Long et al. [76] explored this topic, showing that miR-184 targets EPHX1. Importantly, while the expression of EPHX1 was reduced in HCC tissues compared to adjacent non-tumor tissue, the opposite was observed for miR-184. Additionally, the transfection of miR-184 into HCC cell lines (HepG2 and SMMC-7721) expressing EPHX1 resulted in EPHX1 expression downregulation. Notably, while EPHX1 expression in HCC cell lines downregulated cell proliferation and promoted apoptosis, the opposite occurred for miR-184. Additionally, it was shown that the long non-coding RNA LINC00205 sponges miR-184, thus upregulating EPHX1. In the HepG2 cell line, the silencing of LINC00205 resulted in increased cell proliferation and migration and a reduced apoptotic rate, thus mimicking the effects of miR-184 overexpression. In a xenograft mouse model of HCC, overexpression of EPHX1 decreased tumor growth, while LINC00205 silencing promoted tumor growth, confirming the in vitro data.

The lncRNA beta-secretase 1 antisense RNA (BACE1-AS) has been found to play a negative regulatory role in some tumors, but its implication in HCC is unknown. Tian et al. [77] reported that BACE1-AS is upregulated in HCC tissues (28 HCC samples) comparted to adjacent non-tumor tissue and that a high expression correlated with poor patient prognosis. Moreover, increased serum levels of BACE1-AS were observed in HCC patients compared to healthy individuals. In vitro in HuH6, HuH7, HepG2 and Hep3B HCC cell lines, BACE1-AS was significantly upregulated compared to the normal liver epithelial cell line LO2. In HuH7 and HepG2 HCC cell lines, BACE1-AS silencing downregulated cell proliferation, migration and invasion and promoted apoptosis. Subsequently, the authors identified miR-214-3p as a target of BACE1-AS. It is known that miR-214-3p expression is downregulated in HCC tissues, and that its upregulation induces cell cycle arrest and increases apoptosis in HCC [90]. Thus, BACE1-AS inhibits the anti-tumor effects of miR-214-3p by sponging it. Finally, the authors identified apelin (APLN) as one target of miR-214-3p. APLN expression is increased in HCC tissues and its inhibition downregulates the growth of HCC cells in vitro and in vivo [91]. Despite being of potential interest for novel HCC therapeutic approaches, these interesting data need to be confirmed through an in vivo model of HCC.

### 4.3. circRNAs

All the works presented in this section are summarized in Table 3.

Midkine (MDK) is a basic heparin-binding growth factor with pleiotropic effects including the promotion of cell proliferation, cell migration and angiogenesis. Du et al. [92] demonstrated that the circRNA derived from exon 5 of the MDK gene (circMDK) is upregulated in HCC and correlates with poor patient survival. However, it was not specified whether circMDK is upregulated only in HCC or also in cirrhotic tissue. This is relevant information as most of HCCs occur in cirrhotic liver. Thus, it is not fully clear whether circMDK overexpression is restricted to HCC or it occurs also in cirrhotic tissue. Additionally, the cohort of patients studied (45) needs to be enlarged in the future before drawing a solid conclusion in this regard. From the mechanistic point of view, the increased expression of circMDK resulted in a sponge effect for miR-346 and miR-874-3p. In turn, this led to an increase in the levels of ATG16L1 (autophagy-related 16 like 1), which upregulates the PI3K/AKT/mTOR signaling pathway responsible for the promotion of cell proliferation, migration and invasion. The delivery of chemically synthesized anti-circMDK siRNAs to the HCC-derived cell lines HuH7 and Hep3B resulted in a reduction in cell proliferation, migration and invasion and an increase in cell apoptosis. To test the in vivo effectiveness, the siRNA was delivered with a poly(β-amino esters) (PAE) carrier. This polymer can bind the negatively charged siRNA via electrostatic interactions, thus conferring protection against in vivo degradation. The authors showed that PAE–siRNA inhibited tumor growth in in vivo models of HCC, which included a subcutaneous mouse model, a metastatic model, a patient-derived xenograft model and an orthotopic model of HCC. The authors proposed that PAE–siRNA could passively accumulate into tumor tissues when delivered intravenously. Together, these data support the significance of circMDK targeting in HCC. 

Lin et al. [93] concentrated their attention on circRERE, derived from the RERE gene that encodes the arginine-glutamic acid dipeptide repeat protein. Overexpression of this protein triggers apoptosis. The authors showed that circRERE was overexpressed in the tumor tissues of a cohort of 60 HCC patients. Interestingly, following HCC surgical removal, circRERE expression decreased. This suggests a strong relation of this circRNA with HCC rather than with the HCC surrounding tissue. In in vitro cellular models of HCC (HuH7 and Hep3B cell lines), it was demonstrated that the targeting of circRERE with a siRNA resulted in the weakening of cell viability and invasion and also promoted cell apoptosis. At the molecular level, it was demonstrated that circRERE acts as a sponge for miR-1299, thereby reducing the level of this miR. As a consequence, the gastrulation brain homeobox 2 (GBX2), a target of miR-1299, was upregulated. Notably, the authors demonstrated that GBX2 promotes HCC cell viability and prevents apoptosis. Despite being limited to in vitro models of HCC, the data presented show the therapeutic potential of circRERE targeting and/or miR-1299 upregulation. While the first option seems to be more feasible based on the technology currently available, the upregulation of miR-1299 in HCC tissue seems to be far more complex at the moment.

Synaptophysin-like protein (SYPL1), expressed in different tissues, is a component of transport vesicles. It has also been associated with epithelial-mesenchymal transition and is considered a poor prognostic factor in HCC [105]. Lei et al. [94] examined the expression levels of the circRNA derived from SYPL1 (circSYPL1) in 11 HCC patients, demonstrating that circSYPL1 expression was approximately 1.5-fold higher in HCC tissue compared to the surrounding non-tumor tissue. Moreover, circSYPL1 expression doubled in metastatic tumor tissue compared to non-metastatic tumors. This suggests the relevant contribution of circSYPL1 overexpression in tumor cell migration and invasion. In the HCC cell lines HepG2 and PLC-PRF-5, circSYPL1 silencing by siRNA downregulated cell survival/migration and favored apoptosis. In a xenograft subcutaneous mouse model of HCC generated with PLC-PRF-5 cells, the intratumor injection of a lentivirus expressing the anti circSYPL1siRNA significantly reduced tumor growth. Moreover, in the same group, lung metastasis was reduced compared to the control siRNA-treated animals. At the molecular level, the authors showed that the oncogenic effect of circSYPL1 was due to its sponge effect on miR-506-3p, which targets the protein EZH2. This is a histone-lysine N-methyltransferase enzyme that takes part in histone methylation and, eventually, in transcriptional repression. In tumor cells, EZH2 inhibits the genes responsible for the suppression of tumor development. Thus, circSYPL1 silencing results in an increase in miR-506-3p and a parallel decrease in EXH2. While the in vitro/in vivo data are convincing, the number of patients studied in the work needs to be expanded.

Circular RNA itchy E3 ubiquitin-protein ligase (circ-ITCH) is derived from exons 7–14 of the itchy E3 ubiquitin protein ligase (ITCH) gene. ITCH deficiency causes altered physical growth, defective muscle development and aberrant immune system function [106]. It is known that the expression of circ-ITCH in HCC tissues is reduced and that a high level of circITCH is associated with favorable survival of HCC [107]. Guo et al. [95] explored the molecular mechanisms of the above findings. First, it was confirmed in multiple HCC cell lines (HuH7, HCCLM3, SMMC-7721 and MHCC97H) that circITCH expression is downregulated compared to non-tumor cells (LO2). Moreover, its silencing in HepG2 promoted cell viability, proliferation and migration and reduced apoptosis. Subsequently, it was demonstrated in HCCLM3 and HepG2 that circITCH sponges miR-184. Notably, miR-184 overexpression reversed the anti-tumor effects of circITCH. The anti-HCC role of circITCH was confirmed in vivo using a xenograft mouse model of HCC employing LM3 cells and the same cells stably overexpressing circITCH. The stable expression resulted in a clear reduction in tumor growth and was paralleled by the reduction in miR-184 expression. The target of miR-184 remains to be determined in order to complete the understanding of the pathway.

Multidrug resistance-associated protein 4 (MRP4) is a member of the adenosine triphosphate (ATP)-binding cassette transporter family. It favors the extrusion of different molecules, including drugs, from the cell. Additionally, it is involved in cell communication and drug distribution. It is known that MRP4 is upregulated in HCC tissues [108]; however, the mechanisms behind this phenomenon are not fully clear. Hu et al. [96] studied the possible mechanisms of MRP4 upregulation in the HCC-derived cell lines HuH7 and SMMC-7721. First, the authors confirmed MRP4 upregulation in 19 HCC patients compared to adjacent noncancerous tissues. Subsequently, they showed that miR-124-3p and miR-4524-5p reduced the expression of MRP4 at the protein level. Notably, as no binding site for miR-4524-5p in the MRP4 3’ untranslated region was found, it was postulated that miR-4524-5p regulates MRP4 levels with post-translational mechanisms. It was then proven that both miR-124-3p and miR-4524-5p were sponged by circHIPK3, a circRNA derived from the homeodomain-interacting protein kinase 3 (HIPK3) gene that encodes a serine/threonine-protein kinase involved in the regulation of transcription and apoptosis. The siRNA-mediated knockdown of circHIPK3 resulted in the downregulation of the MRP4 protein by increasing the levels of miR-124-3p and miR-4524-5p. Given the frequent occurrence of drug resistance in HCC, these data are of interest. However, validation in more complex models of HCC is required to fully understand their potential therapeutic value.

Forkhead box protein M1 is a protein encoded by the FOXM1 gene. The FOXM1 protein is a member of the FOX family of transcription factors that promote cell cycle progression [109]. Wang et al. [97] explored the role of circFOXM1 derived from FOZM1 in HCC. The authors showed that the expression of circFOXM1 was increased in different HCC cells (HuH7, HepG2, Hep3B, HCCLM3 and MHCC97-H) compared to normal human hepatocytes (MIHA). The siRNA-mediated silencing of circFOXM1 reduced the proliferation of HuH7 and HepG2 cells as well as migration and invasion. In vivo, in a xenograft mouse model of HCC, the tumor volume and weight of the animal injected with HuH7 overexpressing the siRNA anti circFOXM1 were significantly reduced compared to controls. Additionally, following a database search, the increased expression of circFOXM1 in HCC tissues compared to normal control tissues was observed. With regard to the molecular mechanisms regulating the above observations, the authors demonstrated that miR-1179 expression was significantly upregulated in HuH7 and HepG2 cells after circFOXM1 silencing. This, together with functional assays, indicated that circFOXM1 sponges miR-1179. Subsequently, it was proven that the target of miR-1179 is the mRNA of the sperm-associated antigen 5 (SPAG5). SPAG5 is known to influence the separation of sister chromatids by regulating spindles, thus affecting the cell cycle. Notably, SPAG5 overexpression is associated with lung, breast, cervical and bladder urothelial cancers. By a database search, the authors reported that SPAG5 expression levels inversely correlate with HCC patient overall survival. Together, the data presented indicate that circFOXM1 promotes HCC proliferation and metastasis by downregulating miR-1179 that, in turn, leads to an increase in SPAG5. 

miR-375 is involved in multiple tumors and, in HCC, it inhibits cell growth by targeting ErbB2. The mechanisms of its regulation have been studied by Li et al. [98] in HCC by exploring the possible contribution of the circ_0072088, which is known to act as a sponge of cirRNAs in non-small-cell lung carcinoma. circ_0072088 originates from exon 13 to exon 17 of a protein-coding gene locus named ZFR. In the first step, the authors showed that circ0072088 was upregulated in HCC tissues (49 samples) compared to para-cancerous liver tissue and that patients with larger tumor sizes had increased expression of circ0072088. Moreover, circ0072088 overexpression promoted proliferation and inhibited apoptosis in SNU-398 cells, while its knockdown had the opposite effects. Additionally, circ0072088 overexpression promoted cell migration and invasion. Interestingly, miR-375 expression in SNU-398 cells was repressed following overexpression of circ0072088, while the opposite effect was induced after circ0072088 knockdown in HepG2 cells. Following the demonstration that circ0072088 can bind miR-375, the authors showed that miR-375 inhibited proliferation, migration and invasion and induced apoptosis in HepG2. Then, it was shown that JAK2 (Janus kinase 2), a tyrosine kinase involved in the proliferation of hematopoietic cells, could be a target of miR-375 as it contains the complementary binding site of miR-375. Notably, in HCC tissue, the expression levels of JAK2 positively and negatively correlated with circ_0072088 and miR-375, respectively. Collectively, these data support the concept that circ_0072088 promotes the JAK2 signaling pathway by downregulating miR-375 levels. Further studies in HCC animals are necessary to confirm the interesting data reported.

By both bioinformatics and quantitative real-time PCR, Zhang et al. [99] demonstrated that circC16orf62 expression is increased in HCC tissue (88 paired samples) compared to adjacent non-tumor tissue. Moreover, its upregulation inversely correlates with patient prognosis and directly correlates with tumor diameter and the presence of lymph node metastasis. circC16orf62 overexpression was also proven by the authors in multiple HCC cell lines (BEL7402, QGY7701, SMMC7721, HepG2, Hep3B and HuH7) compared to the non-tumor liver epithelial cell line LO2. In Hep3B and SMMC7721, siRNA-mediated silencing of circC16orf62 resulted in the impairment of cell proliferation, invasion and migration. A downregulation in the expression of aerobic glycolysis enzymes with a metabolic shift towards the anaerobic glycolysis, typical of cancer cells, was also demonstrated. Additionally, it was found that miR-138-5p was negatively correlated with circC16orf62 in HCC tissues and that in HCC cells, circC16orf62 could sponge miR-138-5p. By using prediction software and an experimental approach, it was revealed that protein tyrosine kinase 2 (PTK2) is a target of miR-138-5p. PTK2 is a protein tyrosine kinase concentrated in the focal adhesions and allows cells to attach to extracellular matrix components; in cancer cells it is often related to increased metastatic potential. By using an online database, it was observed that PTK2 was upregulated in HCC tissues. Moreover, in HCC cell lines, the authors observed that miR-138-5p inhibition and PTK2 overexpression could partly reverse the effect of circC16orf62 downregulation. Finally, in a xenograft model of HCC (obtained by Hep3B injection) it was shown that circC16orf62 downregulation retarded tumor growth; however, no data on increased animal survival were shown. Together, these data suggest that circC16orf62 functions as a molecular sponge for miR-138-5p, which in turn promotes PTK2 activity.

The androgen receptor (AR) is a nuclear receptor known to influence HCC progression and its inhibition favors metastasis in advanced stages of HCC. Bao et al. [100] explored the mechanisms of AR involvement in HCC in relation to vasculogenic mimicry (VM). VM contributes to the blood supply to tumors and is based on the formation of neo-vessel lined exclusively in tumor cells, mimicking the presence and function of endothelial cells [110]. Notably, VM is linked to a poor prognosis in HCC [111]. The authors noticed that AR overexpression suppressed VM formation in the HCC cell lines SK and HA22T. At the molecular level, it was observed that AR overexpression in the HCC cell lines SK and HA22T downregulated the levels of circRNA7 that in turn could not sponge miR-7-5p. Targets of miR-7-5p were shown to be VE-cadherin and neurogenic locus notch homolog 4 (Notch4). VE-cadherin is a calcium-dependent cell–cell adhesion glycoprotein indispensable for proper vascular development. Notch4 is a transmembrane protein that regulates the interactions between physically adjacent cells. Thus, miR-7-5p upregulation induced by circRNA7 downregulation impaired the pro-vasculogenic effects of VE-cadherin/Notch4. These results are of particular interest due to the highly vascularized nature of HCC. 

**Figure 6:**
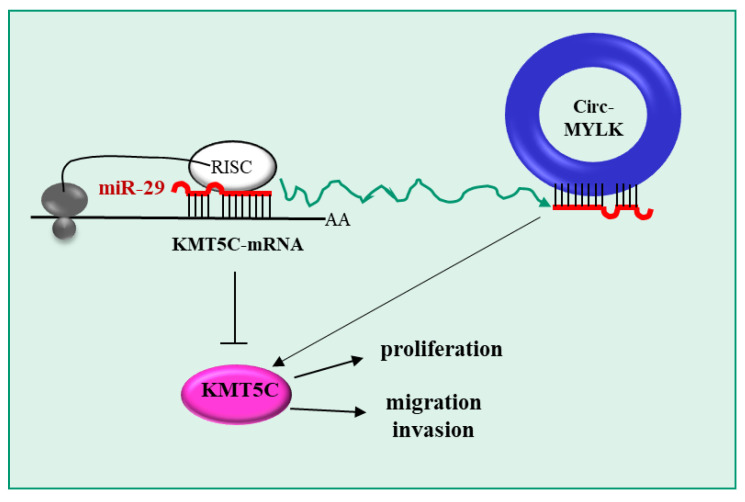
circ-MYLK, Gao et al. [101]. This circular RNA sponges miR-29, which can then no longer target histone lysine N methyltransferase 5C (KMT5C), thus allowing KMTC5 to promote cell proliferation and migration.

MYLK (myosin light chain kinase) is a calcium-/calmodulin-dependent enzyme which phosphorylates the myosin regulatory light chains, allowing myosin interaction with actin filaments to induce muscle contractions. Gao et al. [101] explored the mechanistic role of a circRNA derived from MYLK (circMYLK) in HCC. The rationale for this choice was based on the fact that circMYLK is known to be upregulated in HCC [112]. First, the authors confirmed circMYLK upregulation in 60 HCC tissues compared to adjacent non-tumor areas. Unfortunately, no information about the histology of the control tissues (normal liver, fibrotic and cirrhotic liver) was provided. Despite this, increased circMYLK expression was related to poor overall survival in HCC patients. circMYLK upregulation was confirmed in the HCC cell lines HepG2, PLC, MHCC-97H and HCC-LM3 compared to the control human normal liver cell line HL-7702. circMYLK silencing by a siRNA lead to a decrease in HCC cell proliferation, migration and invasion and an increased rate of apoptosis. Based on a bioinformatic analysis and experimental confirmations, the authors showed that circMYLK sponges miR-29a. This is an interesting observation, as the same authors previously demonstrated that miR-29a was downregulated in HCC and that it could repress HCC cell growth and invasion [113]. It was then found that histone lysine N methyltransferase 5C (KMT5C) is a target gene of miR-29a (Figure 6). Notably, KMT5C regulates the transcription and maintenance of genome integrity and it is known to be correlated with the progression of gastrointestinal tumors [114]. Moreover, the authors showed that KMT5C was overexpressed in HCC tissue samples and that KMT5C was negatively correlated with miR-29a expression and positively correlated with circMYLK levels. Finally, KMT5C silencing downregulated HCC cell growth, migration and invasion. The potential therapeutic value of circMYLK silencing by siRNA was also proven in a subcutaneous mouse model of HCC, obtained by injecting the HCC-LM3 cell line.

Pu et al. [102] explored the role of circ-0000092 in HCC starting from the knowledge that it is involved in HCC metastasis and tumorigenesis [115]. An in silico analysis predicted that miR-338-3p could interact with circ-0000092 and that hematopoietic- and neurologic-expressed sequence 1 (HN1) might be a target gene of miR-338-3p. Notably, HN1 correlates with a poor prognosis in HCC patients and its knockdown reduces cell growth and migration [116]. In 40 HCC tissue samples, the authors observed increased and decreased expressions of circ-0000092 and miR-338-3p, respectively, compared to adjacent normal tissues. These data were confirmed in vitro in the HCC cell lines Hep3B, LM3, MHCC97L, SK-hep1 and HepG2 compared to the normal liver cell line THLE-2. Moreover, an increased expression was observed for HN1 in HCC tissue samples compared to adjacent normal tissue. Subsequently, it was proven that circ-0000092 sponges miR-338-3p and that this results in the promotion of cell proliferation, migration, invasion and angiogenesis for LM3 and SK-hep1 cell lines. This phenomenon was accompanied by a concomitant increase in HN1 levels secondary to the decrease in miR-338-3p. In a xenograft mouse model of HCC generated with the LM3 cell line, it was observed that circ-0000092 silencing resulted in a reduction in tumor volume and weight, which was paralleled by a decrease in the expression of HN1. Thus, circ-0000092 and/or HN1 targeting has the potential to be of therapeutic value in HCC.

The circRNA of microtubule-associated serine/threonine kinase 1 (MAST1), or circMAST1, is derived from exons 9–11 of MAST1, which encodes a protein essential for correct brain development. Yu et al. [103] showed in 15 HCC tissue samples that circMAST1 expression was increased compared to that of the matching adjacent normal liver tissues. Interestingly, the authors also showed that the serum levels of circMAST1 were higher in patients with HCC compared to healthy controls, opening the way for a possible employment of a liquid biopsy as diagnostic tool. In the HCC cell lines HCCLM3 and HepG2, silencing circMAST1 increased the proportions of cells in the G0/G1 phase while decreasing G2 phase cells. Notably, this was paralleled by a decrease in the levels of the cell cycle promoter cyclin E (essential for the G1/S phase transition), cyclin A (essential for the S phase) and the cyclin-dependent kinases (CDKs) 1 and 2 (important for both the G1 and S phases). Moreover, circMAST1 silencing suppressed the migration and invasion of HCCLM3 and HepG2 cell lines. In a xenograft mouse model of HCC (obtained by grafting the HCCLM3 cell line), it was confirmed that the silencing of circMAST1 resulted in decreased tumor volumes and weights; this was paralleled by a decrease in cyclin E/cyclin A/CDKs. In silico predictions, together with experimental confirmations, indicated that miR-1299 was sponged by circMAST1. Notably, miR-1299 is known to be a tumor suppressor in HCC and the author also demonstrated that circMAST1 upregulates cell growth and invasion via miR-1299 downregulation. Interestingly, it was demonstrated that one target of miR-1299 is catenin beta-1 (CTNNB1), a protein involved in the regulation and coordination of cell–cell adhesion and gene transcription. Moreover, CTNN1 upregulation represents one of the major molecular alterations in HCC [117]. The fact that circMAST1 expression positively correlates with CTNND1 expression suggests the functional role of the circMAST1–miR-1299–CTNND1 pathway in promoting HCC and the potential therapeutic value of its inhibition.

Previous data have revealed that a circular RNA derived from the superoxide dismutase 2 mitochondrial (SOD2) gene (circ-SOD2) is overexpressed in HCC tumor tissues compared to adjacent normal liver tissues [118]. SOD2 is a member of the iron/manganese superoxide dismutase that eliminates mitochondrial reactive oxygen species (ROS) and thus protects the cell from death. Zhao et al. [104] confirmed circ-SOD2 overexpression in 18 out of 19 HCC patient’s tumor tissues, also showing that the expression levels inversely correlated with patient prognosis. circ-SOD2 overexpression was then proven in the liver cancer cell lines HepG2, HuH7, SK-HEP1 and HEP3B compared to the normal liver cell HL-7702. The authors demonstrated that circ-SOD2 can sponge miR-502-5p and that in both HCC liver tissues and HCC cell lines, the levels of circ-SOD2 and miR-502-5p are inversely correlated. Additionally, circSOD2 overexpression promotes in vitro (HepG2 and HuH7) cell proliferation and in vivo tumorigenesis (xenograft mouse model generated with HepG2). DNMT3a, a DNA methyl transferase particularly relevant for establishing DNA methylation patterns during development, was found to be a target for miR-502-5p. In adult cells, DNMT3a activates the JAK2/STAT3 (Janus kinases/signal transducer and activator of transcription proteins 3) signaling pathway by suppressing SOCS3 (suppressor of cytokine signaling 3), the inhibitor of JAK2/STAT3. Upregulation of JAK2/STAT3 is connected to cancer development. Finally, it was shown that STAT3 can upregulate circ-SOD2 expression in a positive feedback fashion. Together, these data indicate that circ-SOD2 targeting may be of therapeutic value as it would result in the inhibition of the pro-tumorigenic pathway miR-502-5p/ DNMT3a/ JAK2/STAT3. 

Finally, Lyu et al. [63] have shown that circWHSC1 expression is elevated in HCC tissues and cells and that this is associated with worse overall survival in HCC patients. Notably, knockdown of circWHSC1 diminished HCC cell proliferation and metastasis in vitro and downregulated tumorigenesis in vivo. From the mechanistic point of view, the authors showed that circWHSC1 acts as a sponge for miR-142-3p, a relevant miRNA in HCC (Zeng et al. [49]). In turn, a reduction in miR-142-3p levels increased the amount of one of its targets, i.e., homeobox A1 (HOXA1). This target belongs to the family of transcription factors that regulate gene expression, morphogenesis and cellular differentiation. This work, together with those of Zeng et al. [49] and Cui et al. [64], points towards the relevance of miR-142-3p in downregulating HCC.

### 4.4. ncRNAs and Drug Resistance

In the attempt to improve the effectiveness of the available anti-HCC drugs, many works have explored the mechanisms of drug resistance development in HCC. Here, in particular, we focus on those based on non-coding RNAs. All the works presented in this section are summarized in Table 4.

By a database search and experimental tests in 90 HCC tissues compared to paired non-tumor tissue, Lu et al. [119] observed that miR-23a-3p was upregulated in HCC tissues and that upregulation was inversely correlated with patient survival. Additionally, patients with higher miR-23a-3p levels showed a worse progression-free survival following sorafenib treatment. In a subcutaneous xenograft mouse model of HCC resistant to sorafenib (generated by MHCC97L cell line), it was shown that miR-23a-3p expression was increased ten-fold in HCC tissue compared to non-tumor tissue. In an orthotopic model of HCC generated with miR-23a-3p knockout MHCC97L cells, it was observed that while miR-23a-3p knockout cells had a slightly delay in tumor growth, their growth was dramatically decreased when mice received sorafenib. This was mainly due to an increase in a subtype of apoptosis named ferroptosis. This is an iron-dependent apoptosis characterized by the accumulation of peroxidized lipid products and iron overload, known to contribute to sorafenib resistance in HCC [126]. Acyl-CoA synthetase long-chain family member 4 (ACSL4) is an enzyme necessary for catalyzing lipid peroxidation to trigger ferroptosis. Upon treatment with sorafenib, the authors observed that an increased expression of miR-23a-3p was responsible for the downregulation of ACSL4 with a consequent decrease in cell death by ferroptosis. These data suggest that sorafenib effectiveness may be improved by the contemporary targeting of miR-23a-3p by siRNA.

**Figure 7:**
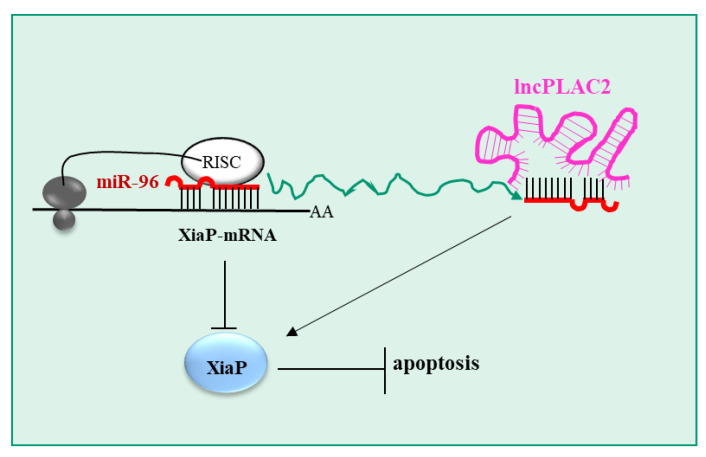
lncPLAC2, Wang et al. [120]. This lncRNA sponges miR-96, thus preventing its inhibitory effect on the X-linked inhibitor of apoptosis protein (XiaP), a known anti-apoptotic protein. This mechanism contributes to explaining the induction of resistance against the drug cisplatin.

The lncRNA placenta-specific protein 2 (PLAC2) was shown to be upregulated (1,73-old) by Wang et al. [120] in the HCC tissues of 62 patients. No significant correlations with patient age, gender, tumor size, number and differentiation stages were observed. Notably, it was reported that PLCA2 plasma levels increased at 0, 2 and 4 months following cisplatin administration. This observation was confirmed also in cultured HCC cell lines SNU-475 and SNU-387 following cisplatin treatment. At the molecular level, the authors showed in in vitro models that the increased PLAC2 levels resulted in the sponging of miR-96. In turn, decreased miR-96 levels allowed an increase in the expression of its target X-linked inhibitor of apoptosis protein (XiaP) (Figure 7). XiaP is known to protect cells from apoptosis, thereby contributing to cancer development and drug resistance. Although they are of potential interest, these data need to be corroborated by additional evidence about the relevance of miR-96 and XIAP in HCC, i.e., by showing their upregulation in patients following cisplatin treatment.

Chen et al. [121] reported that the sensitivity of the HCC cell lines HuH7, Hep3B, SNU-387 and SNU-449 to sorafenib treatment was inversely correlated with the expression levels of lncRNA-POIR (human periodontal ligament stem cell osteogenesis impairment-related lncRNA). The siRNA-mediated knockdown of lncRNA-POIR increased the sensitivity to sorafenib compared to control siRNA-treated cells. By informatic analyses, the authors showed that miR-182-5p was the downstream target of lncRNA-POIR. Experimentally, it was shown that the levels of lncRNA-POIR and miR-182-5p were inversely correlated in HCC cell lines. Functional studies have also revealed that lncRNA-POIR acts as a sponge for miR-182-5p. The upregulation of miR-182-5p led to a strong increase in E-cadherin expression with the complementary downregulation of vimentin expression, events known to prevent EMT. The authors thus proposed that miR-182-5p can improve sorafenib sensitivity by preventing EMT. The direct interaction of miR-182-5p with vimentin was however not provided.

The role of EMT in sorafenib resistance was also studied by Hirao et al. [122]. miRNA microarray analyses performed in resistant HCC cell lines (PLC/PRF5-R1 and PLC/PRF5-R2) and subsequent confirmation assays showed that miR-125b-5p was upregulated. Additionally, miR-125b-5p transfection into sorafenib-sensitive cells (PLC/PRF5) significantly increased the drug resistance. This was paralleled by a decrease in E-cadherin and an increase in vimentin levels, thus indicating a shift towards EMT. It was then shown that a possible target of miR-125b-5p was ataxin-1 (ATXN1). This is a ubiquitous protein predominantly expressed in the cell nucleus, where it interacts with chromatin and several transcriptional repressors. The inhibition of ATXN1 resulted in the activation of SNAIL (zinc finger protein SNAI1), which is known to repress the expression of the adhesion molecule E-cadherin, thus favoring EMT. Moreover, miR-125b-5p promoted cell migration and invasion via the inhibition of ATXN1. Finally, in a subcutaneous mouse model of HCC generated with PLC/PRF5 cells stably expressing miR-125b-5p, treatment with sorafenib was definitively less effective in terms of tumor growth inhibition compared to the control. Importantly, the authors showed that in patients with low ATXN1 expression, the overall survival was much shorter than in the high-expressing ATXN1 patients.

HOX transcript antisense intergenic RNA (HOTAIR) is a 2,158 nucleotide long lncRNA, localized between HOXC11 and HOXC12, both encoding transcription factors. HOTAIR has been reported to be aberrantly expressed in several tumors, including gastrointestinal tumors and HCC [127]. Tang et al. [123] observed that sorafenib resistance was increased in HCC cells (HuH7, Hep3B, SNU-387 and SNU-449) overexpressing HOTAIR compared to the normal hepatic cell line LO2. Moreover, HOTAIR silencing resulted in an increased sensitivity to sorafenib. This was paralleled by an increase in E-cadherin and a decrease in vimentin expression, indicating a block of EMT. The authors also showed that HOTAIR sponges miR-217 and that miR-217 overexpression improved cell sensitivity to sorafenib. As observed following HOTAIR silencing, miR-217 overexpression resulted in an increase in E-cadherin and a decrease in vimentin expression. This work, which needs to be confirmed in a more complex HCC model, further supports the concept that sorafenib resistance depends on EMT.

Xu et al. [124] investigated the role of lncRNA H19 in sorafenib resistance. The rationale for the choice of lncRNA H19 was that it contributes to the resistance to 5-fluorouracil (5-FU) in colorectal cancer. In 18 HCC patient tissues, the authors observed increased H19 expressions compared to matched normal liver tissue. This observation was confirmed in the HCC cell lines SNU-387, SNU-449, HuH7 and Hep3B. The highest H19 expression was observed in SNU-387 and SNU-449, while it was somewhat reduced in HuH7 and Hep3B. Interestingly, cells with higher H19 expression were more resistant to sorafenib, indicating a negative relation between sorafenib sensitivity and H19 expression. In line with this, H19 silencing improved HCC cells’ sensitivity to sorafenib. Moreover, H19 silencing reduced HCC cell invasion and migration. At the molecular level, this was paralleled by a decrease in vimentin and an increase in E-cadherin expression, indicating a block of EMT. The authors also observed that H19 upregulates miR-675 expression, without, however, providing any mechanistic explanation. Despite this, it was shown that miR-675 upregulation in SNU-387, SNU-449, HuH7 and Hep3B increased cell resistance to sorafenib and promoted EMT. More mechanistic knowledge is required to better understand the role of H19/miR-675 to promote EMT and thus sorafenib resistance.

Nucleus accumbens-associated protein-1 (NACC-1) is a transcriptional regulator implicated in the induction of tumorigenesis in ovarian and breast cancers. Yin et al. [125] showed that in HCC tissues (20 samples), NACC-1 is overexpressed compared to adjacent non-tumorous tissues. NACC-1 silencing in the HCC cell lines HepG2, HuH7 and HCC-1 resulted in a marked decrease in cell proliferation and colony formation migration and invasion. Moreover, NACC-1 silencing increased the sensitivity to the anticancer drug doxorubicin. In particular, cells were more prone to doxorubicin-induced apoptosis. By bioinformatics and experimental tests, the authors proved that NACC-1 is the target of miR-760. Importantly, miR-760 was downregulated in HCC tissues compared to adjacent non-tumorous tissues and an inverse correlation with NACC-1 expression was proven. Moreover, in vitro, the overexpression of miR-760 reduced cell migration and invasion. While further testing of in vivo models is necessary to substantiate the data presented, it would be interesting to deepen the understanding of the mechanisms regulating NACC-1-induced resistance to doxorubicin.

### 4.5. ncRNAs in Liver Fibrosis and HCC

All the works presented in this section are summarized in Table 5.

**Figure 8:**
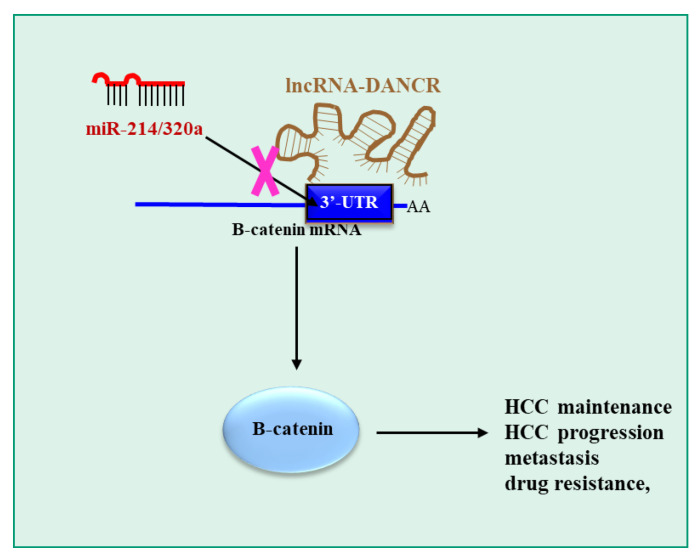
lncDANCR, Gan et al. [128]. By interacting with the 3’-untranslated region (3’-UTR) of the β-catenin mRNA, lncDANCR prevents the binding of miR214/320a. This results in the upregulation of the pro-tumorigenic protein β-catenin. This mechanism may be also at the base of fibrosis induction in the liver.

It has been previously demonstrated [132] that the expression level of lncRNA differentiation antagonizing non-protein coding RNA (lncRNA-DANCR) is increased in HCC. Moreover, it binds to the 3′ untranslated region of β-catenin (CTNNB1), blocking its degradation by miR-214 and miR-320a (Figure 8). β-catenin regulates and coordinates cell-cell adhesion and gene transcription; moreover, it is involved in HCC maintenance, drug resistance, tumor progression and metastasis [133]. Recently, Gan et al. [128] explored the role of lncRNA-DANCR in LF. The authors observed that lncRNA-DANCR expression corresponds to the severity of fibrosis in a carbon tetrachloride (CCl_4_)-induced mouse model. Interestingly, it was shown that lncRNA-DANCR was also increased in animal serum, opening the possibility to consider this molecule a marker of LF. Moreover, in lncRNA-DANCR knockout animals, LF was less evident following CCl_4_ treatment compared to control animals, underlining the role of lncRNA-DANCR in promoting LF. The authors also observed that HCC rate formation was reduced, thus suggesting lncRNA-DANCR as a common molecular promoter for both HCC and LF. Finally, the silencing of lncRNA-DANCR in a patient-derived xenograft mouse model of HCC confirmed the reduced occurrence and development of HCC. This elegant work clearly demonstrates the molecular connection between HCC and LF and opens the possibility for a common therapeutic target.

It has been observed that the knockdown of miR-124 is related to poor survival in HCC patients [134]. Moreover, miR-124 is known to limit renal fibrosis [135]. Based on this evidence, Yang et al. [129] explored the contribution of miR-124 in LF. In a CCl_4_-induced liver fibrosis mice model, the authors showed that miR-124 was downregulated, particularly in HSCs [9], the liver cell type mostly involved in LF. In parallel, the pro-inflammatory cytokines TNF-α, IL-6 and IL-1B were upregulated. The fact that miR-124 upregulation induced a decrease in the levels of the above cytokines suggested a molecular connection between these molecules. By bioinformatics and experimental conformation in HSC, it was found that miR-124 targets the mRNA of IQ motifs, including guanosine triphosphatase-activating protein 1 (IQGAP1). IQGAP1 modulates cell architecture and cytoskeletal rearrangements; moreover, it is overexpressed in many cancer cell lines and has the potential function of modulating fibrosis diseases [136]. Both in vivo in a CCl_4_-induced LF model and in vitro in HSC, it was observed that IQGAP1 was upregulated and that it promoted the expression and secretion of TNF-α, IL-6 and IL-1B, thus triggering LF. This interesting paper further points towards a molecular connection between LF and HCC.

The activation of HSCs not only generates LF, but it can also promote HCC [8]. Notably, HSC activation also depends on immune cells. In this regard, interleukin-17 (IL-17)-producing T helper (CD4+) cells (Th17) are known to participate in the formation of the tumor microenvironment (TME) [137]. Thus, Feng et al. [130] explored the role of Th17 (CD4+/IL17+) in the activation of HSCs. The authors concentrated on the effects of miR-132, as this miR was known to correlate to Th17 cell differentiation and disease progression. First, the authors demonstrated that HCC-derived Th17 (CD4+/IL17+) cells express higher levels of miR-132 compared to Th (CD4+), which do not express IL17 (IL17-). In cultured cells, miR-132 overexpression was proven to increase IL17 and IL22 expressions. By bioinformatic analyses and confirmation by experiments, it was shown that smad nuclear-interacting protein 1 (SNIP1) was a possible target for miR-132. SNIP1 is a transcription regulator that plays a role in several intracellular signaling pathways such as cell growth and fibrogenesis. SNIP1 was directly connected to IL17/IL22 expression as its silencing resulted in an increase in the two cytokines. The authors showed that the overlay of HSCs with conditioned media from Th17 overexpressing miR-132 (and thus IL17/IL22) resulted in an increased expression of α-SMA and vimentin, two markers of HSC activation. Moreover, activated HSCs promoted HCC and EMT, as shown by the decreased expression of E-cadherin and the increased migration. This work underlines the relevance of immune cells in the genesis of both LF and HCC, a fact that deserves attention for the development of novel therapeutic approaches.

Dong et al. [131] revealed that miR-369 expression is downregulated both in LF (20 patients) and in HCC (120 patients) compared to normal liver tissue (obtained from patients who underwent surgical resection for hepatic hemangioma; the distal para-hemangioma tissues were chosen, 10 patients). Moreover, in HCC, it was observed that in recurrence HCC tissue, miR-369 levels were significantly lower than in the primary tumor tissues and that miR-369 levels were directly correlated with patient prognosis. In HCCLM3 and HuH7 cell lines, miR-369 overexpression reduced HCC cell growth and colony formation. Additionally, cell migration and invasion were downregulated. These data were confirmed in a xenograft mouse model of HCC (generated inoculating HCCLM3 subcutaneously), where, in addition to a delay in tumor growth, a decrease in lung metastasis was observed. Notably, the authors also showed a decreased expression of vimentin and an increase in E-cadherin, thus indicating the inhibition of EMT. At the molecular level, it was demonstrated that the target of miR-369 was the zinc finger E-box binding homeobox 1 (ZEB1), which belongs to a family of transcription factors known to promote EMT. Notably, ZEB1 silencing resulted in reduced HCC cell growth and migration/invasion. While the authors deeply explored the role of miR-369 in HCC, limited information was provided for LF. In this regard, it would have been interesting to know whether miR-369 expression is downregulated in HSC, the major factor responsible for LF, and if miR-369 downregulation could activate HSC, thus promoting LF. 

## 5. Conclusions

Despite the significant therapeutic improvements obtained in the field of HCC in recent years, patient prognosis remains poor due to late diagnosis, chemotherapy failure, frequent recurrence and the lack of effective molecular targets. The papers presented in this review indicate that targeting of ncRNAs has the potential to downregulate HCC with several different mechanisms. However, some considerations should be made in this regard. 

First, there are many different ncRNAs associated with HCC, suggesting that the regulation of HCC is more complex than we would like it to be. In this regard, an aspect that deserves further investigation is the possibility that ncRNA expression may vary during the course of the disease, as it does for other oncogenic molecules in cancer. Thus, it is possible that for effective approaches, different ncRNAs need to be targeted in the course of the disease to combat disease recurrence. Additionally, for effective HCC downregulation, it may be reasonable to complementarily target multiple ncRNAs. Which ncRNAs to target may depend on the specific expressions in a given patient, thus realizing the concept of precision/personalized medicine. 

A second aspect to be considered is the complex interplay among different ncRNAs. For example, the anti-oncogenic miR-138-5p is sponged by both lncRNA UPK1A-AS1 [72] and circC16orf62 [99]. This implies that the silencing of UPK1A-AS1 and circC16orf62 should probably be pursued at the same time. However, it is not known whether UPK1A-AS1 and circC16orf62 are always upregulated in the same patients, and we do not know the consequences of targeting a ncRNA that is not upregulated in HCC. Another example of the complex regulation of an anti-oncogenic miRNA is that of miR-142-3p, which is sponged by circWHSC1 [63] and lnc712 [64]. An additional example is miR-1299, which is sponged by both circRERE [93] and circMAST1 [103]. Obviously, for these last two examples, the considerations made for miR-138-5p hold true and further highlight the intricate regulatory network of ncRNAs in HCC. 

Third, the papers presented here invariably indicate that the possibility to target oncogenic ncRNAs is at the moment restricted to the use of molecules such as siRNAs that interact with ncRNAs via specific base pairings. However, siRNAs suffer from poor stability in the biological environment and thus need appropriate delivery systems to make them effective. The identification of optimized delivery systems is not an easy task due to the number of variables in effective HCC targeting. For example, the identification of specific cancer-related antigens can be very complex, since the surface antigens of tumor cells may change over time. Thus, the optimization of an anti-ncRNA therapeutic approach must also take into consideration the issue of delivery of the therapeutic molecules.

Despite the above considerations, we believe that future HCC therapeutic approaches should combine the targeting of specific surface cancer antigens with the use of molecules (such as siRNAs and also other RNA molecules which could be of interest [138,139,140]) that are able to hit targets predominantly overexpressed in cancer cells compared to the normal cells. We are confident that, by considering the above aspects together with the encouraging works described here, ncRNA targeting may result in the development of effective anti-HCC therapies in the near future.

## Figures and Tables

**Table 1:** miRNA involvement in hepatocellular carcinoma.

miRNA	miRNA Target *	Reference
miR-129-5p (*Ts*)	SOX4/HDGF	[46]
miR-139-5p (*Ts*)	ROCK2	[47]
miR-137 (*Ts*)	Notch-1	[48]
miR-142-3p (*Ts*)	PIK3CG	[49]
miR-221 (*O*)	Not indicated	[50]
miR-106a-5p (*O*)	PTPN12	[51]
miR-29b-3p (*Ts*)	Not indicated	[52]
miR-495 (*Ts*)	CTRP3	[53]
miR-23a-3p (*O*)	PCDH17	[54]
miR-126-5p (*Ts*)	EGFR	[55]
miR-642a (*O*)	SEMA4C	[56]

*Ts*: tumor suppressor; *O*: oncogenic. * SOX4: SRY-related HMG-box; HDGF: hepatoma-derived growth factor; ROCK2: Rho-associated coiled coil kinase-2; PIK3CG: phosphatidylinositol-4,5-bisphosphate 3-kinase catalytic subunit gamma isoform; PTPN12: protein tyrosine phosphatase non-receptor type 12; CTRP3: C1q tumor necrosis factor (TNF)-related proteins; PCDH17: protocadherin17; EGFR: epidermal growth factor receptor; SEMA4C: semaphorin 4C.

**Table 2:** lncRNAs in hepatocellular carcinoma.

lncRNA *	miRNA	miRNA Target *	Reference
lnc712 (*O*)	miR-142-3p-*Ts*	Bach-1	[64]
lncRNA ASAP1-IT1 (*O*)	miR-221-3p-*Ts*	Not indicated	[67]
LINC00152 (*O*)	miR143a-3p-*Ts*	KLC2	[68]
lncRNA CASC9 (*O*)	miR-424-5p-*Ts*	Not indicated	[69]
LINC00839 (*O*)	miR-144-3p-*Ts*	WTAP	[70]
lncPVT1 (*O*)	miR-214-*Ts*	GDF15	[71]
lncUPK1A-AS1 (*O*)	miR-138-5p-*Ts*	CDK6	[72]
lnc MALAT1 (*O*)	miR-375-*Ts*	YAP	[73]
lnc linc00467 (*O*)	miR-18a-5p-*Ts*	NEDD9	[74]
lnc LINC00958-*O* (*O*)	miR-3619-5p-*Ts*	HDGF	[75]
lnc LINC00205 (*Ts*)	miR-184-*O*	EPHX1	[76]
lnc BACE1-AS (*O*)	miR-214-3p-*Ts*	APLN	[77]

*Ts*: tumor suppressor; *O*: oncogenic. * ASAP1-IT1: the intronic transcript 1 (IT-1) of ArfGAP with SH3 domain, ankyrin repeat and PH domain 1 (ASAP1); LINC00152: long intergenic non-coding RNA 152; KCL2: kinesin light chain 2; CASC9: cancer sensitivity 9; WTAP: WT1-associated protein; lncPVT1: plasmacytoma variant translocation 1; UPK1A-AS1: UPK1A antisense RNA 1; CDK6: cyclin-dependent kinase 6; MALAT1: metastasis-associated lung adenocarcinoma transcript 1; YAP: yes-associated protein 1; NEDD9: neural precursor cell expressed developmentally downregulated protein 9; HDGF: hepatoma-derived growth factor; BACE1-AS: beta-secretase 1 antisense RNA; APLN: apelin.

**Table 3:** circRNAs involvement in hepatocellular carcinoma.

circRNA *	Sponged miRNA	miRNA Target *	Reference
circMDK (*O*)	miR-346, miR-874-3p-*Ts*	ATG19L1	[92]
circRERE (*O*)	miR-1299-*Ts*	GBX2	[93]
circSYPL1 (*O*)	miR-506-3p-*Ts*	EZH2	[94]
circITCH (*Ts*)	miR-184-*O*	Not indicated	[95]
circHIPK3 (*O*)	miR-124-3p, miR-4524-5p-*Ts*	MRP4	[96]
circFOXM1 (*O*)	miR-1179-*Ts*	SPAG5	[97]
circ0072088 (*O*)	miR-375-*Ts*	JAK2	[98]
circC16orf62 (*O*)	miR-138-5p-*Ts*	PTK2	[99]
circRNA7 (*O*)	miR-7-5p-*Ts*	VEcadherin/Notch	[100]
circMYLK (*O*)	miR-29a-*Ts*	KMT5C	[101]
circ0000092 (*O*)	miR-338-3p-*Ts*	HN1	[102]
circMAST1 (*O*)	miR-1299-*Ts*	CTNNB1	[103]
circSOD2 (*O*)	miR-502-5p-*Ts*	DNMT3a	[104]
CircWHSC1 (*O*)	miR-142-3-*Ts*	HOXA1	[63]

*Ts*: tumor suppressor; *O*: oncogenic. * MDK: midkine; ATG16L1 (autophagy-related 16 like 1); RERE: arginine-glutamic acid dipeptide repeats protein; GBX2: gastrulation brain homeobox 2; SYPL1: synaptophysin-like protein; EXH2: enhancer of zeste homolog 2; ITCH: itchy E3 ubiquitin protein ligase; HIPK3: homeodomain-interacting protein kinase 3; MRP4: multidrug resistance-associated protein 4; FOXM1: forkhead box protein M1; SPAG5: sperm-associated antigen 5; JAK2: Janus kinase 2; PTK2: protein tyrosine kinase 2; MYLK: myosin light chain kinase; KMT5C: histone lysine N methyltransferase 5C; HN1: hematopoietic- and neurologic-expressed sequence 1; MAST1: microtubule-associated serine/threonine kinase 1; CTNNB1: catenin beta-1; SOD2: superoxide dismutase 2, mitochondrial; DNMT3a: DNA methyl transferase3a; HOXA1: homeobox A1.

**Table 4:** ncRNAs and drug resistance in hepatocellular carcinoma.

lncRNA *	miRNA	miRNA Target *	Reference
	miR-23a-3p (*O*)	ACSL4	[119]
lncRNA-PLAC2 (*O*)	miR-96 (*Ts*)	XiaP	[120]
lncRNA-POIR (*O*)	miR-182-5p (*Ts*)	Vimentin?	[121]
	miR-125b-5p (*O*)	ATXN1	[122]
lncRNA-HOTAIR (*O*)	miR-217 (*Ts*)	Vimentin?	[123]
lncRNA-H19 (*O*)	miR-675 (*O*)	Not indicated	[124]
	miR-760 (*Ts*)	NACC-1	[125]

*Ts*: tumor suppressor; *O*: oncogenic. * ACSL4: acyl-CoA synthetase long-chain family member 4; PLAC2: placenta-specific protein 2; XiaP: X-linked inhibitor of apoptosis protein; POIR: human periodontal ligament stem cell osteogenesis impairment; ATXN1: ataxin-1; HOTAIR: HOX transcript antisense intergenic RNA; NACC1: nucleus accumbens-associated protein-1.

**Table 5:** ncRNAs and liver fibrosis/hepatocellular carcinoma.

lncRNA *	miRNA	miRNA Target *	Reference
lncRNA-DANCR (*O*)	miR-214, miR-320a (*Ts*)	CTNNB1	[128]
	miR-124 (*O*)	IQGAP1	[129]
	miR-132	SNIP1	[130]
	miR-369 (*Ts*)	ZEB1	[131]

*Ts*: tumor suppressor; *O*: oncogenic. * DANCR: differentiation antagonizing non-protein coding RNA; IQGAP1: IQ motif including guanosine triphosphatase-activating protein 1; SNIP1: smad nuclear interacting protein 1; ZEB1: zinc finger E-box binding homeobox 1 CTNNB1: catenin beta-1.

## Data Availability

Not applicable.
